# Neuronal Intranuclear Inclusion Disease with *NOTCH2NLC* GGC Repeat Expansion: A Systematic Review and Challenges of Phenotypic Characterization

**DOI:** 10.14336/AD.2024.0131-1

**Published:** 2024-01-31

**Authors:** Tian Zeng, Yiqun Chen, Honghao Huang, Shengqi Li, Jiaqi Huang, Haobo Xie, Shenyi Lin, Siyao Chen, Guangyong Chen, Dehao Yang

**Affiliations:** ^1^The First School of Medicine, School of Information and Engineering, Wenzhou Medical University, Wenzhou, China.; ^2^Department of Cardiology, Renji Hospital, Shanghai Jiao Tong University School of Medicine, Shanghai, China.; ^3^Department of Neurology, The Third Affiliated Hospital of Wenzhou Medical University, Wenzhou, China.; ^4^Department of Neurology, The Second Affiliated Hospital, Zhejiang University School of Medicine, Hangzhou, China.

**Keywords:** *NOTCH2NLC*, neuronal intranuclear inclusion disease, systematic review

## Abstract

Neuronal intranuclear inclusion disease (NIID) is a highly clinically heterogeneous neurodegenerative disorder primarily attributed to abnormal GGC repeat expansions in the *NOTCH2NLC* gene. This study aims to comprehensively explore its phenotypic characteristics and genotype-phenotype correlation. A literature search was conducted in PubMed, Embase, and the Cochrane Library from September 1, 2019, to December 31, 2022, encompassing reported NIID cases confirmed by pathogenic *NOTCH2NLC* mutations. Linear regressions and trend analyses were performed. Analyzing 635 cases from 85 included studies revealed that familial cases exhibited significantly larger GGC repeat expansions than sporadic cases (*p* < 0.001), and this frequency significantly increased with expanding GGC repeats (*p* trend < 0.001). Age at onset (AAO) showed a negative correlation with GGC repeat expansions (*p* < 0.001). The predominant initial symptoms included tremor (31.70%), cognitive impairment (14.12%), and muscle weakness (10.66%). The decreased or absent tendon reflex (DTR/ATR) emerged as a notable clinical indicator of NIID due to its high prevalence. U-fiber was observed in 79.11% of patients, particularly prominent in paroxysmal disease-dominant (87.50%) and dementia-dominant cases (81.08%). Peripheral neuropathy-dominant cases exhibited larger GGC repeat expansions (median = 123.00) and an earlier AAO (median = 33.00) than other phenotypes. Moreover, a significant genetic anticipation of 3.5 years was observed (*p* = 0.039). This study provides a comprehensive and up-to-date compilation of genotypic and phenotypic information on NIID since the identification of the causative gene *NOTCH2NLC*. We contribute a novel diagnostic framework for NIID to support clinical practice.

## INTRODUCTION

Neuronal intranuclear inclusion disease (NIID) is a genetically confirmed neurodegenerative disorder known for its significant clinical heterogeneity, affecting multiple systems, and characterized by the presence of eosinophilic intranuclear inclusions [1]. In 1968, Lindenberg et al. initially reported a case with atypical widespread nuclear inclusion bodies [2]. Subsequently, Sung et al. described a case featuring intranuclear hyaline inclusions in neurons associated with the atrophy of multiple systems, coining the term “neuronal intranuclear hyaline inclusion disease” in 1980 [3]. In 1984, Haltia et al. officially termed the condition “neuronal intranuclear inclusion disease” in a case report [4]. In 2011, skin biopsy emerged as a valuable and safe alternative to rectal and sural nerve biopsy for NIID diagnosis [5], significantly enhancing pathology confirmation and aiding in disease identification. Later, in 2016, Sone et al. documented a high-intensity signal in the corticomedullary junction through diffusion-weighted imaging (DWI) in cranial magnetic resonance imaging (MRI), commonly referred to as “U-fiber”, providing a valuable diagnostic clue [6, 7]. Despite these advancements, the high clinical heterogeneity of NIID poses significant challenges in its identification. Its clinical symptoms encompass cognitive impairment [8], resting tremor [9], muscle weakness [10], autonomic nervous system dysfunction [11], encephalitic episodes [12], and others. This, combined with a lack of awareness among physicians, had led to frequent misdiagnoses and delayed recognition of NIID patients in the past.

In 2019, researchers in Japan and China successively established GGC repeat expansions in the 5' UTR of the *NOTCH2NLC* gene as the causative mutation [13-16]. The revelation sparked massive interest in the field of neuroscience, leading to numerous investigations into NIID. Consequently, an increasing number of cases initially diagnosed with amyotrophic lateral sclerosis (ALS), multiple system atrophy (MSA), Parkinson's disease (PD), and others have been reported to be associated with NIID [17-19]. These findings have significantly broadened the spectrum of the clinical phenotypes of NIID and facilitated the retrospective recognition of previously misdiagnosed patients. Indeed, the misdiagnosis of NIID as other neurodegenerative disorders, such as PD, can have detrimental consequences for patient care. While symptomatic treatments like levodopa may alleviate specific symptoms (e.g., bradykinesia) in misdiagnosed NIID cases, they fail to address the underlying genetic cause and offer no curative potential [20].

Owing to its pronounced clinical heterogeneity and limitations in the current diagnostic process, the prevalence of NIID in the Eastern Asian population might be significantly underestimated. Furthermore, the recent surge in NIID researches, while valuable, has resulted in diverse descriptions and categorizations of symptoms and phenotypes in reports, potentially causing confusion and misinterpretations for readers. Given these challenges, an updated review of NIID is essential to establish a scientific classification of NIID symptoms and phenotypes. This would provide standardized expressions for efficient screening and diagnosis while fostering further exploration into the pathogenesis and identification of corresponding therapy targets.

Our study compiled publications from the identification of the causative gene up to 2022. We comprehensively summarized clinical characteristics, neuroimaging features, electrophysiological characteristics, pathological characteristics, and phenotype-genotype relations in genetically confirmed NIID patients. Building on these findings, we proposed a schematic diagnostic framework for NIID to assist the clinical diagnostic process. In summary, this research aims to offer crucial information for expanding current knowledge and exploring future possibilities in the understanding and management of NIID.

## MATERIALS AND METHODS

### Search strategy and study selection

We conducted a thorough search of all publications in PubMed, Embase, and the Cochrane Library from September 1, 2019, to December 31, 2022, utilizing the search term “*NOTCH2NLC* OR (notch 2 N-terminal like C)”. Publications were considered eligible and initially included if they met pre-determined inclusion criteria: studies reporting at least one individual screening positive for pathogenic *NOTCH2NLC* GGC repeat expansions (GGC repeat expansions > 40 [21] and/or confirmed by the original author), regardless of study designs. After removing duplicates, two reviewers (ZT and CYQ) independently filtered the records based on article contents by labeling them as ‘include’, ‘exclude’ or ‘maybe’. Additionally, we checked relevant publications and reference lists for articles that might have been missed with the aforementioned approach. After completion of the screening process, the blind mode was turned off and conflicts were resolved by consensus with a third reviewer (YDH).

### Quality assessment and data extraction

Quality assessment and data extraction for each selected study were independently conducted by two reviewers (ZT and CYQ) and subsequently revised by a third reviewer (YDH). Study quality was appraised based on a modified quality score scale described by Anne Grünewald et al. [22], encompassing six domains: sequence analysis, imaging examination, pathological test, nerve conduction study (NCS), demographic data, and clinical assessment. The score distribution for sequence analysis is as follows: 0 points for studies not reporting GGC repeat expansions, 1 point for those reporting unspecified number of GGC repeat expansions (e.g., only reporting a range), and 2 points for those reporting precise numbers. In the other five domains, scores of 0 or 1 were assigned based on the absence or presence of relevant data. The highest score of quality assessment for each article is 7 points.

Meanwhile, we extracted data pertaining to the characteristics of the studies and the study population: author, year of publication, number of patients screened, demographic details, family history, age at onset (AAO), disease durations. Other important variables, including GGC repeat expansions in *NOTCH2NLC*, clinical manifestations, cranial MRI results, NCS findings, and pathological test outcomes for each index patient were extracted as well. Individuals with GGC repeat expansions in *NOTCH2NLC* fewer than 40 were regarded as normal and excluded from the records. Familial status was determined if more than one patient in the same family exhibited similar symptoms, as defined by the original studies. Disease duration less than 1 year was recorded as 0 years.

Clinical manifestations collected could be categorized into following groups: 1) cognitive impairment/dementia, abnormal behavior, and mental /psychiatric symptoms; 2) peripheral neuropathy including muscle weakness, sensory disturbance, and decreased/absent tendon reflex (DTR/ATR); 3) tremor; 4) autonomic dysfunction, encompassing bladder dysfunction, sexual dysfunction, constipation, diarrhea, hyperhidrosis, orthostatic hypotension, miosis, and unreasoned vomiting; 5) neurological attacks, such as disturbance of consciousness, seizure, stroke-like episodes, and encephalitic episodes; 6) parkinsonism; 7) other symptoms like ataxia, dysarthria, dysphagia, cough and headache, etc. [6, 15, 23, 24]. Phenotypes of individuals reported in original articles were also extracted and could be mainly divided into four categories: movement disorders, peripheral neuropathy, dementia, and paroxysmal disease. Pathological tests were deemed positive if any of the following criteria were met: 1) the presence of neuronal intranuclear inclusion; 2) ubiquitin-positive; 3) p62-positive.

During this process, we identified patients reported multiple times by the same research groups, and such patients were included only once with information merged. Finally, the extracted data were tabulated into a datasheet for further analysis.

### Missing data management and statistical analyses

Unless explicitly stated, we assumed that individuals were from the same geographical countries as the corresponding authors of the original articles. In analyzing clinical manifestations, unreported symptoms in certain cases were considered “absent” if the corresponding clinical symptoms were reported in other individuals. This approach was exclusively applied to case series meticulously conducted and comprehensively reported by the original authors to mitigate potential biases caused by incomplete reporting. A similar methodology was employed to analyze cranial MRI results, NCS findings and pathological test outcomes.

Statistical analyses were conducted using SPSS Statistics 25.0 software and Medcalc 22.0. Continuous variables were expressed as median (interquartile range [IQR]), and categorical variables were presented as frequencies. Mann-Whitney U tests and Kruskal-Wallis tests were performed to assess the significance of differences between groups for continuous variables, while categorical variables were analyzed using χ2 tests or Fisher's exact tests. Simple linear regression was employed to explore the relationship between AAO and GGC repeat expansions. Jonckheere-Terpstra trend tests and trend χ′ tests were carried out for trend analysis. A *p* value < 0.05 was regarded as statistically significant.

**Figure 1.**
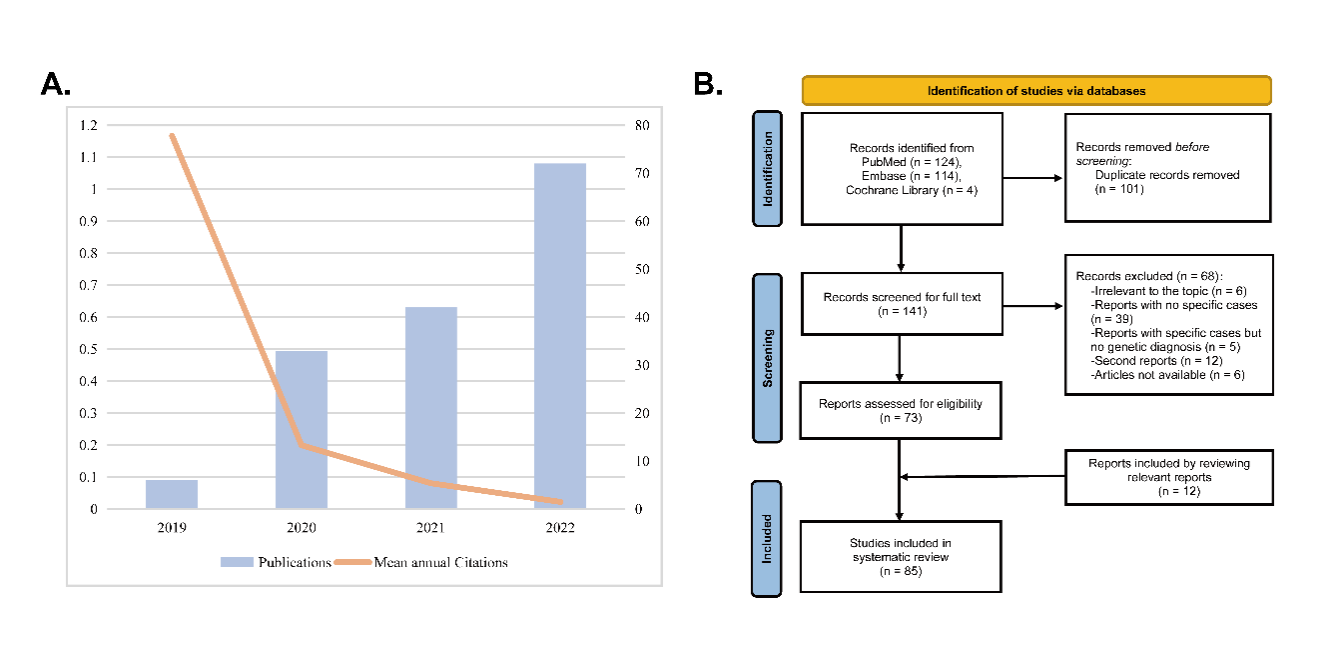
Summary of the number of relevant studies and mean annual citations from 2019 to 2022 (**A**) and flowchart for literatures identification and selection (**B**).

**Figure 2.**
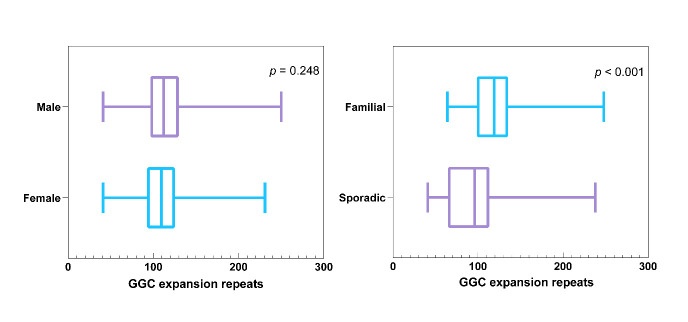
**Boxplots showing the differences of GGC repeat expansions in male and female cases (**A**) and in familial and sporadic cases (**B**)**. Notes: (**A**) There was no significant difference in GGC repeat expansions between male and female (*p* = 0.248); (**B**) GGC repeat expansions in familial cases were significantly larger than sporadic cases (*p* < 0.001).

## RESULTS

### Literature search and study identification

A total of 242 studies were initially identified in the literature search. To provide a brief overview of the progress in this field, the number of studies and mean annual citations from September 1, 2019, to December 31, 2022, were displayed in Figure 1A. The number of published studies peaked in 2022, while mean annual citations peaked in 2019, indicating a potential mismatch between research output and immediate recognition.

Among the 242 identified studies, 101 duplicate records were removed. Further exclusions encompassed reports deemed irrelevant to the topic, second reports such as replies and errata, studies lacking specific cases or genetic diagnosis, as well as those with unavailable full contents. Figure 1B provides a summary of the study identification and screening procedure. Ultimately, 85 studies meeting the inclusion criteria were included in this review, and their characteristics and quality assessment scores are provided in Supplementary Table 1.

**Table 1:** Summary of characteristics of patients with tremors, cognitive impairment and muscle weakness.

	Tremor	Non-tremor	*p*
**No. of cases (%)**	110 (31.70)	237 (68.30)	
**AAO, median (IQR)**	53.00 (43.00-59.00) [99]	55.00 (44.00-62.00) [215]	0.041
**Familial, No. (%)**	19 (40.43) [47]	57 (37.50) [152]	0.718
**Male, No. (%)**	45 (43.27) [104]	104 (45.41) [229]	0.715
**GGC repeat numbers, median (IQR)**	110.00 (90.00-125.00) [97]	110.00 (91.00-128.00) [199]	0.355
	Cognitive impairment	Non- cognitive impairment	*p*
**No. of cases (%)**	49 (14.12)	298 (85.88)	
**AAO, median (IQR)**	62.00 (58.00-67.00) [46]	53.00 (41.00-60.00) [268]	< 0.001
**Familial, No. (%)**	6 (22.22) [27]	70 (40.70) [172]	0.066
**Male, No. (%)**	21 (42.86) [49]	128 (45.07) [284]	0.774
**GGC repeat numbers, median (IQR)**	110.00 (97.00-123.00) [39]	110.00 (90.50-128.00) [257]	0.982
	Muscle weakness	Non- muscle weakness	*p*
**No. of cases (%)**	37 (10.66)	310 (89.34)	
**AAO, median (IQR)**	32.00 (26.00-54.50) [37]	55.00 (48.00-62.00) [277]	< 0.001
**Familial, No. (%)**	21 (60.00) [35]	55 (33.54) [164]	0.003
**Male, No. (%)**	22 (59.46) [37]	127 (42.91) [296]	0.056
**GGC repeat numbers, median (IQR)**	124.50 (89.00-168.75) [26]	109.50 (91.00-125.00) [270]	0.061

Abbreviations: AAO: age at onset. The number in [] represents the actual number of people with avaible records.

**Figure 3.**
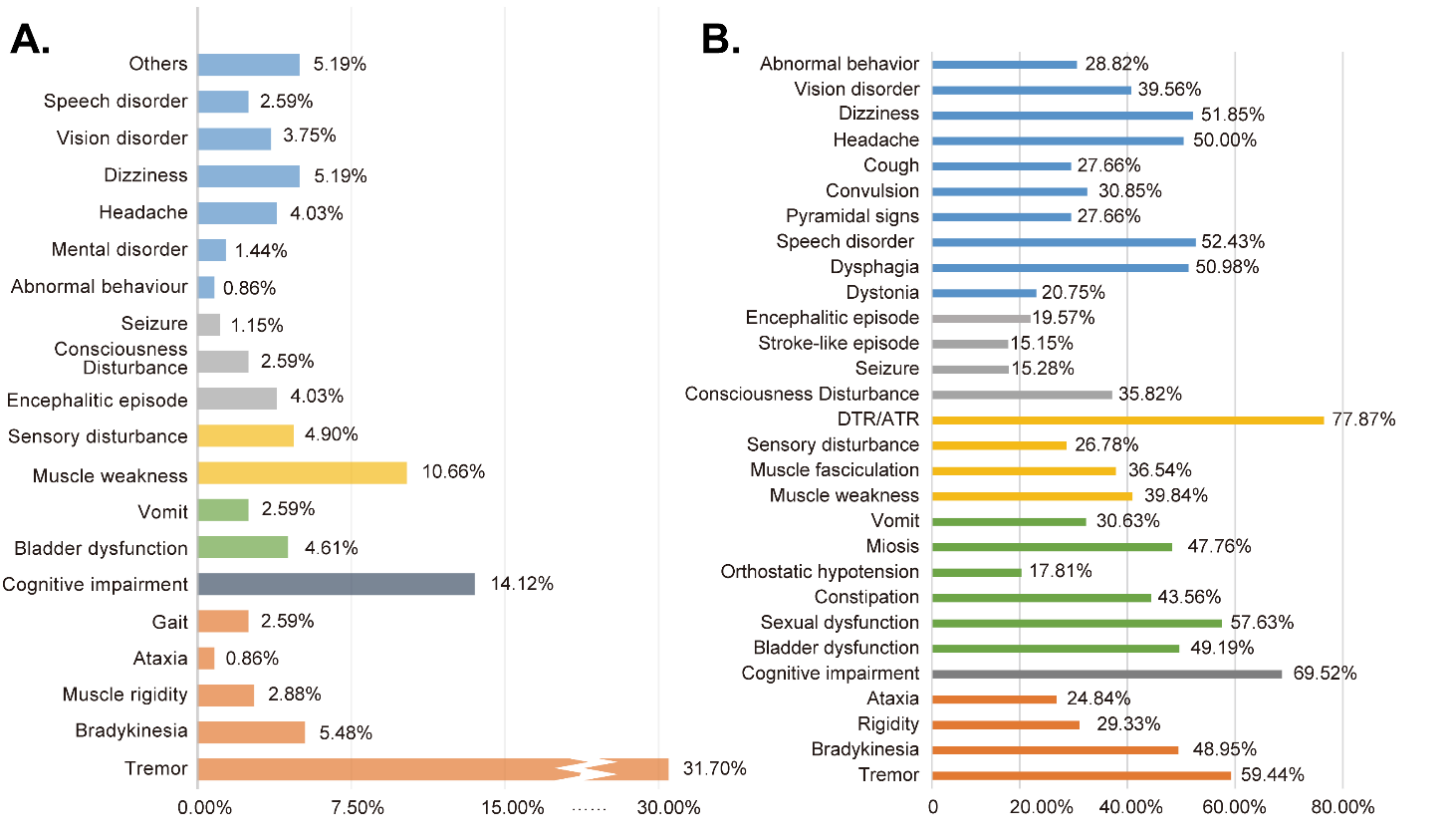
**Bar chart presenting the frequencies of initial symptoms (**A**) and present clinical symptoms and signs of NIID patients (**B**)**. Notes: (**A**) The three most common initial symptoms were tremor (31.70%), cognitive impairment (14.12%) and muscle weakness (10.66%); (**B**) the three most common symptoms were cognitive impairment (69.52%), tremor (59.44%) and sexual dysfunction (57.63%). Abbreviations: DTR/ATR: decreased or absent tendon reflex.

### Overview of genetically confirmed NIID patients included in the study

Data were extracted from 635 genetically confirmed cases, excluding 20 duplicate cases reported in 4 studies. There were 297 index cases, including probands from 71 families and 226 sporadic cases. Most index cases originated from China (75.3%), followed by Japan (23.0%). AAO was available for 500 cases (median: 54.00, IQR: 42.00-62.00, range: 1-82), while age at research (AAR) was available for 480 cases (median:63.00, IQR: 54.00-69.00, range: 13-91). Males constituted 46.8% of the 564 cases for which gender data was reported.

**Table 2:** Summary of clinical characteristics of patients with different dominant phenotypes.

	Movement Disorders	Peripheral Neuropathy	Dementia	Paroxysmal Disease	*p*
**No. of cases**	141	76	57	43	
**AAO, median (IQR)**	53.00 (43.50- 60.00) [105]	33.00 (25.25-46.75) [64]	60.00 (56.50-65.00) [49]	56.00 (51.00-65.00) [31]	*p* < 0.001
**AAR, median (IQR)**	63.00 (55.00-68.00) [103]	48.00 (40.25-58.75) [72]	66.50 (63.00-72.25) [42]	64.00 (60.00-68.50) [41]	*p* < 0.001
**Duration, median (IQR)**	10.00 (4.00-15.00) [103]	10.00 (3.00-18.00) [63]	7.00 (2.00-12.00) [43]	6.00 (2.00-7.00) [30]	*p* = 0.007
**Familial (%)**	50.57 (44/87)	64.06 (41/64)	63.83 (30/47)	14.81 (4/27)	*p* < 0.001
**Male (%)**	48.39 (60/124)	60.34 (35/58)	42.11 (24/57)	34.88 (15/43)	*p* = 0.063
**Country**					
**China**	94.33 (133/141)	61.84 (47/76)	78.95 (45/57)	88.37 (38/43)	
**Japan**	1.42 (2/141)	38.16 (29/76)	19.30 (11/57)	6.98 (3/43)	
**Others**	4.26 (6/141)	0 (0/76)	1.75 (1/57)	4.65 (2/43)	
**GGC repeat numbers, median (IQR)**	103.50 (63.50-120.00) [106]	123.00 (104.00-148.00) [43]	106.50(91.75-122.75) [38]	102.00 (89.50-115.75) [36]	*p* < 0.001
**Tremor (%)**	93.85 (122/130)	40.00 (24/60)	50.00 (21/42)	39.13 (9/23)	*p* < 0.001
**Bradykinesia (%)**	59.42 (41/69)	23.81 (5/21)	36.00 (9/25)	25.00 (1/4)	*p* = 0.010
**Rigidity (%)**	82.76 (24/29)	3.45 (1/29)	14.29 (3/21)	0.00 (0/3)	*p* < 0.001
**Ataxia (%)**	16.18 (11/68)	18.00 (9/50)	10.81 (4/37)	25.00 (4/16)	*p* = 0.589
**Cognitive impairment (%)**	25.64 (20/78)	25.00 (8/32)	100.00 (55/55)	65.22 (15/23)	*p* < 0.001
**Bladder dysfunction (%)**	34.29 (12/35)	46.15 (18/39)	65.71 (23/35)	40.00 (6/15)	*p* = 0.058
**Constipation (%)**	73.68 (14/19)	46.15 (6/13)	55.56 (5/9)	50.00 (1/2)	*p* = 0.410
**Orthostatic hypotension (%)**	7.14 (1/14)	33.33 (4/12)	25.00 (2/8)	0.00 (0/2)	*P* = 0.379
**Miosis (%)**	11.54 (3/26)	20.00 (5/25)	36.36 (8/22)	20.00 (3/15)	*p* = 0.238
**Vomit (%)**	0.00 (0/5)	50.00 (8/16)	8.33 (1/12)	44.44 (12/27)	*p* = 0.028
**Muscle weakness (%)**	25.00 (18/72)	79.45 (58/73)	29.73 (11/37)	38.46 (10/26)	*p* < 0.001
**Muscle fasciculation (%)**	0.00 (0/18)	73.33 (11/15)	0.00 (0/1)	0.00 (0/0)	*p* < 0.001
**Sensory disturbance (%)**	8.82 (6/68)	49.09 (27/55)	24.32 (9/37)	23.08 (6/26)	*p* < 0.001
**DTR/ATR (%)**	88.24 (15/17)	89.66 (26/29)	45.45 (5/11)	81.25 (13/16)	*p* = 0.021
**Consciousness disturbance (%)**	8.47 (5/59)	19.23 (5/26)	25.00 (7/28)	66.67 (12/18)	*p* < 0.001
**Seizure (%)**	3.23 (1/31)	25.00 (1/4)	11.11 (1/9)	42.11 (8/19)	*p* = 0.003
**Stroke-like episode (%)**	7.41 (2/27)	8.70 (2/23)	8.33 (2/24)	31.25 (5/16)	*p* = 0.144
**Encephalitic episode (%)**	0.00 (0/29)	4.00 (1/25)	5.71 (2/35)	70.37 (19/27)	*p* < 0.001
**Dystonia (%)**	4.55 (1/22)	60.00 (3/5)	0.00 (0/7)	18.18 (2/11)	*p* = 0.017
**Dysphagia (%)**	33.33 (1/3)	51.43 (18/35)	0.00 (0/0)	100.00 (1/1)	*p* = 1.000
**Dysarthria (%)**	28.57 (2/7)	86.71 (18/21)	0.00 (0/0)	55.56 (10/18)	*p* = 0.013
**Pyramidal signs (%)**	6.67 (1/15)	77.78 (7/9)	0 (0/1)	33.33 (1/3)	*p* < 0.001
**Convulsion (%)**	72.73 (8/11)	26.67 (4/15)	25.00 (1/4)	100.00 (1/1)	*p* = 0.039
**Cough (%)**	0.00 (0/1)	50.00 (1/2)	0.00 (0/1)	0.00 (0/1)	*p* = 1.000
**Headache (%)**	0.00 (0/5)	40.00 (2/5)	14.29 (1/7)	62.96 (17/27)	*p* = 0.011
**Dizziness (%)**	25.00 (4/16)	0.00 (0/4)	37.50 (3/8)	53.85 (7/13)	*p* = 0.205
**Vision (%)**	0.00 (0/4)	52.94 (9/17)	23.53 (4/17)	35.00 (7/20)	*p* = 0.153
**Abnormal behavior (%)**	0.00 (0/25)	9.68 (3/31)	54.84 (17/31)	33.33 (4/12)	*p* < 0.001

Abbreviations: AAO: age at onset; AAR: age at research; DTR/ATR: decreased or absent tendon reflex. The number in [] represents the actual number of people with avaible records.

Out of a total of 414 cases with available specific GGC repeats records, 9 harbored GGC repeat expansions > 300 (Supplementary Table 2), which were considered non-pathogenic by Deng et al. [25]. More information on these patients is displayed in the Supplementary Results. Among the remaining cases, the median GGC repeat number was 108.00 (IQR: 91.00-125.00, range: 41-250). There was no significant difference in GGC repeat expansions between males and females (median: 112.00 [n = 154] vs 109.50 [n = 184], *p* = 0.248) (Fig. 2). Familial NIID cases exhibited significantly larger GGC repeat expansions compared to sporadic cases (median: 119.00 [n = 87] vs 96.00 [n = 139], *p* < 0.001) (Fig. 2). Thirty-five cases displayed intermediate-length GGC repeat expansions (median: 46.00, IQR: 43.00-50.00).

### Clinical manifestations of NIID patients at onset time and examination

Initial clinical manifestations were documented in 347 patients, with the three most common initial symptoms being tremor (31.70%), cognitive impairment (14.12%) and muscle weakness (10.66%) (Fig. 3A). AAO differed based on initial symptoms: tremor (median: 53 years), cognitive impairment (median: 62 years), muscle weakness (median: 32 years) (Table 1). Interestingly, individuals with onset symptoms of muscle weakness were more likely to have a positive family history than those without (60.00% vs 33.54%, *p* = 0.003). In Figure 3B, symptoms and signs at examination were clustered using different colors, and their frequencies were summarized. Notably, DTR/ATR, previously documented as an important clinical clue [26, 27], was prominent in the majority of patients (77.87%, 95/122). A list of data sources of clinical manifestations can be found in supplementary materials (Supplementary Table 5).

### Classifying NIID patients into four phenotypes based on clinical manifestations

Phenotypes frequently reported included movement disorders, peripheral neuropathy, dementia, and paroxysmal disease [6, 23]. We identified 141, 76, 57 and 43 patients with phenotypes of movement disorders, peripheral neuropathy, dementia, and paroxysmal disease, respectively (Table 2). Within the movement disorders-phenotype, tremor was the most common symptom (122 out of 130 available records), followed by rigidity and bradykinesia. Surprisingly, 73.68% (14/19) of patients had constipation, a common symptom of autonomic dysfunction. DTR/ATR, as mentioned before, remained prominent in this phenotype (88.24%, 15/17) (Table 2).

**Figure 4.**
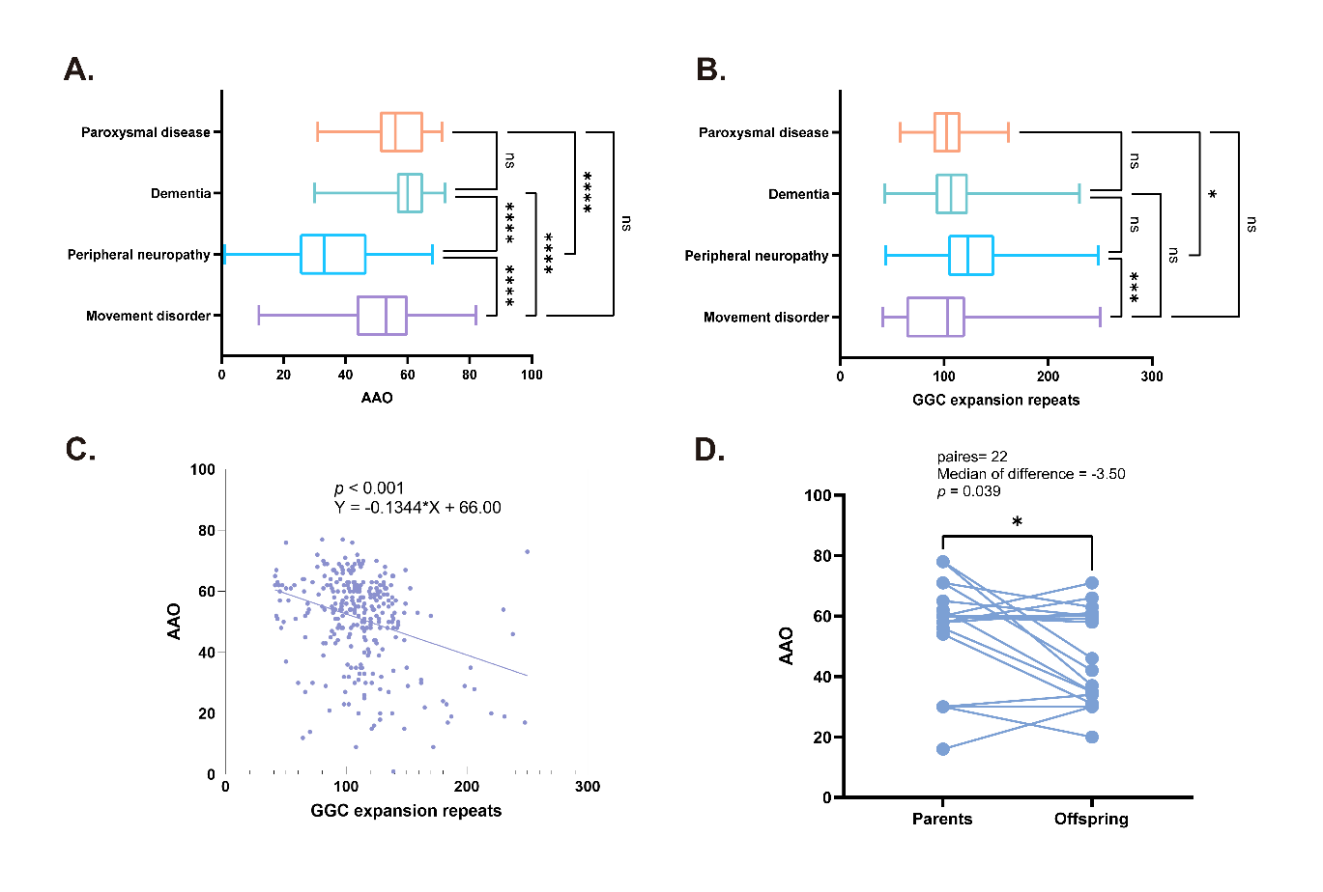
**Genotype-phenotype relations: boxplots comparing the differences of AAO (**A**) and GGC repeat expansions (**B**) in different dominant phenotypes; linear regression investigating the correlation between AAO and GGC repeat expansions (**C**); paired comparison of AAO between offspring and parents (**D**)**. Notes: (**A**) The AAO in peripheral neuropathy-phenotype (median: 33.00) was significantly earlier than patients in any other phenotypes (*p* < 0.001); (**B**) the GGC repeat expansion in neuropathy-dominant patients was relatively larger than that in movement disorders-dominant patients and paroxysmal disease-dominant patients (median: 123.00 vs 103.50, *p* < 0.001; median: 123.00 vs 102.00, *p* = 0.034); (**C**) a significant negative linear correlation was observed (Y = -0.1344*X + 66.00, *p* < 0.001); (**D**) Twenty-two pairs of parent-offspring with available AAO were recognized and significant genetic anticipation was present in these pedigrees (*p* = 0.039). ns: *p* ≥ 0.05, *: *p* < 0.05, ***: *p* ≤ 0.01, ****: *p* ≤ 0.001.

For peripheral neuropathy patients, an earlier AAO (median: 33.00) was a notable feature (Fig. 4A). 64.06% of these cases were familial. Dysarthria (86.71%), muscle weakness (79.45%) and muscle fasciculation (73.33%) were common symptoms. Additionally, DTR/ATR occurred in nearly 90% of patients (Table 2).

Dementia-dominant patients typically experienced onset around their 60s (median: 60.00, IQR: 56.50-65.00). They exhibited cognitive impairment and various clinical symptoms, including bladder dysfunction (65.71%), constipation (55.56%), and abnormal behavior (54.84%). The proportion of patients presenting DTR/ATR (45.45%, 5/11) was significantly lower than other phenotypes (*p* = 0.021) (Table 2).

Among patients with a paroxysmal disease phenotype, only 4 out of 27 cases were familial. They experienced the shortest diseases durations (median: 6.00, IQR: 2.00-7.00) and presented with a high prevalence of consciousness disturbance (66.67%, 12/18) and cognitive impairment (65.22%, 15/23) (Table 2).

### Exploration of neuroimaging, electrophysiological, and pathological characteristics

Clinical examinations conducted on genetically confirmed NIID patients are summarized in Table 3. U-fiber, recognized as a diagnostic sign of NIID, was observed in 250 out of 316 patients, particularly prominent in those suffering from paroxysmal disease and dementia (87.50%, 28/32; 81.08%, 30/37). Another typical radiological sign, leukoencephalopathy, was found in 82.64% of total cases and in every case with the phenotype of paroxysmal disease. Cerebral atrophy and ventricular distension, indicating age-related biochemical degenerations or disease-related pathological changes, were also frequently observed in cases with available records (61.36%, 54/88; 74.78%, 86/115), particularly prominent in dementia-dominant patients (Table 3). 78.38% (29/37) of cases presented FLAIR high-intensity signals in the corpus callosum.

Regarding electrophysiological features, motor nerve conduction velocity (MCV) and sensory nerve conduction velocity (SCV) slowing were present in a large proportion of cases (85.23%, 150/176 and 77.11%, 128/166), while compound muscle action potential (CMAP) and sensory nerve action potential (SNAP) reduction were less frequently detected (Table 3). Notably, approximately 80% of patients dominated by peripheral neuropathy presented with CMAP reduction, which was more remarkable than other phenotypes (*p* = 0.001). In addition, even in patients without symptoms of muscle weakness and sensory disturbance (n = 177), the frequency of MCV slowing and SCV slowing remained high, at 77.66% (73/94) and 69.05% (58/84) respectively, suggesting the existence of subclinical neuropathy.

**Table 3:** Summary of clinical examinations of patients.

	Total	Movement Disorders	Peripheral Neuropathy	Dementia	Paroxysmal Disease	*p*
**No. of cases**	626	141	76	57	43	
**MRI Tests**						
**U-fiber**	79.11 (250/316)	44.00 (22/50)	43.48 (10/23)	81.08 (30/37)	87.50 (28/32)	*p* < 0.001
**Leukoencephalopathy**	82.64 (200/242)	47.62 (20/42)	58.62 (17/29)	86.36 (19/22)	100.00 (14/14)	*p* < 0.001
**FLAIR high-intensity signals in corpus callosum**	78.38 (29/37)	50.00 (3/6)	71.43 (5/7)	100.00 (3/3)	100.00 (3/3)	*p* = 0.505
**Cerebral atrophy**	61.36 (54/88)	34.78 (8/23)	60.00 (6/10)	91.67 (11/12)	86.67 (13/15)	*p* = 0.001
**Ventricular distension**	74.78 (86/115)	27.78 (5/18)	54.55 (6/11)	83.33 (10/12)	75.00 (6/8)	*p* = 0.014
**Nerve conductions**						
**MCV slowing**	85.23 (150/176)	90.48 (19/21)	94.44 (34/36)	80.00 (16/20)	82.35 (14/17)	*p =* 0.321
**SCV slowing**	77.11 (128/166)	60.00 (12/20)	84.21 (32/38)	75.00 (15/20)	75.00 (12/16)	*p =* 0.238
**CMAP reduction**	50.62 (82/162)	60.00 (12/20)	82.35 (28/34)	45.00 (9/20)	21.43 (3/14)	*p* = 0.001
**SNAP reduction**	42.57 (63/148)	60.00 (12/20)	60.61 (20/33)	40.00 (8/20)	38.46 (5/13)	*p =* 0.308
**MMSE**	25.00 (20.00-28.00) [234]	26.00 (23.00-28.00) [83]	28.00 (27.00-29.50) [21]	15.00 (10.00-22.50) [21]	25.50 (20.50-28.75) [12]	*p* < 0.001
**MoCA**	20.00 (13.00-25.00) [73]	19.00 (16.00-24.50) [17]	25.00 (20.75-26.00) [14]	9.00 (5.25-24.50) [8]	25.00 (16.00-29.00) [7]	*p =* 0.053
**Pathological Tests**	98.31 (349/355)	93.10 (27/29)	98.00 (49/50)	95.12 (39/41)	100.00 (32/32)	*p =* 0.416

Abbreviations: MCV: motor nerve conduction velocity; SCV: sensory nerve conduction velocity; CMAP: compound muscle action potential; SNAP: sensory nerve action potential. The number in [] represents the actual number of people with avaible records.

Pathological tests were positive in 349 out of 355 cases (98.31%) (Table 3). A summary of the 6 patients with negative pathological results is displayed in Supplementary Table 3, with relevant results provided in Supplementary Results.

We further explored the association between genotype and phenotype in NIID patients with identified gene mutations. Out of the 414 available records of GGC repeat expansions, only 370 records were matched to their corresponding index cases. We found an increased GGC repeat number in peripheral neuropathy-dominant patients compared to other phenotypes (median: 123.00, IQR: 104.00-148.00) (Fig. 4B). To better understand the relationship between phenotype and genotype, we stratified patients with available GGC repeat expansions into three tertiles (tertile 1: GGC repeats 41-97, n = 124; tertile 2: GGC repeats 98-120, n = 127; tertile 3: GGC repeats 121-250, n = 119) and subsequently performed trend analyses (Table 4).

**Table 4:** Genotype-phenotype relationships.

	Unavailable GGC repeats	Tertile 1 (GGC repeats 41-97)	Tertile 2 (GGC repeats 98-120)	Tertile 3 (GGC repeats 121-250)	*P* trend
**AAO, median (IQR)**	55.00 (35.00-63.00) [190]	56.50 (50.50-64.00) [96]	55.00 (45.50-61.00) [112]	51.50 (31.50-59.00) [100]	*p* < 0.001
**AAR, median (IQR)**	65.00 (56.00-70.00) [207]	64.00 (57.00-68.00) [94]	63.00 (56.25-68.00) [91]	58.00 (45.50-67.00) [79]	*p* = 0.004
**Duration, median (IQR)**	9.00 (4.00-18.00) [190]	5.00 (1.00-10.00) [84]	7.00 (3.00-12.00) [83]	9.00 (5.00-13.75) [71]	*p* < 0.001
**Familial, No. (%)**	63.20 (146/231)	21.65 (21/97)	41.67 (25/60)	59.42 (41/69)	*p* < 0.001
**Male, No. (%)**	47.25 (103/218)	44.66 (46/103)	42.62 (52/122)	49.56 (56/113)	*p* = 0.457
**Tremor (%)**	49.66 (72/145)	68.54 (61/89)	64.71 (66/102)	60.22 (56/93)	*p* = 0.241
**Cognitive impairment (%)**	91.00 (91/100)	55.56 (55/99)	69.61 (71/102)	61.46 (59/96)	*p* = 0.386
**Muscle weakness (%)**	41.13 (51/124)	44.44 (36/81)	29.41 (25/85)	43.82 (39/89)	*p* = 0.983
**Consciousness disturbance (%)**	33.00 (33/100)	29.27 (12/41)	32.73 (18/55)	16.36 (9/55)	*p* = 0.120
**DTR/ATR (%)**	76.00 (19/25)	70.83 (17/24)	72.22 (26/36)	89.19 (33/37)	*p* = 0.067
**MRI Tests**					
**U-fiber (%)**	88.46 (138/156)	62.07 (36/58)	82.35 (42/51)	66.67 (34/51)	*p* = 0.544
**Leukoencephalopathy (%)**	79.07 (68/86)	71.74 (33/46)	91.49 (43/47)	88.89 (56/63)	*p* = 0.021
**Cerebral atrophy (%)**	60.00 (6/10)	45.24 (19/42)	94.74 (18/19)	64.71 (11/17)	*p* = 0.034
**Nerve conductions**					
**MCV slowing (%)**	89.87 (71/79)	80.65 (25/31)	75.76 (25/33)	87.88 (29/33)	*p* = 0.446
**SCV slowing (%)**	74.67 (56/75)	76.00 (19/25)	78.79 (26/33)	81.82 (27/33)	*p* = 0.587
**Pathological Tests (%)**	99.41 (169/170)	98.31 (58/59)	100.00 (61/61)	93.85 (61/65)	*p* = 0.116

Notes: 370 patients with available GGC repeat expansions were categorized into tertiles (n = 124, 127, 119) according to GGC repeats number and trend analyses were performed. The medians for tertiles were 81, 110, 134, respectively. 256 patients with unavailable GGC repeat expansions were also presented. Abbreviations: AAO: age at onset; AAR: age at research; DTR/ATR: decreased or absent tendon reflex. MCV: motor nerve conduction velocity; SCV: sensory nerve conduction velocity. The number in [] represents the actual number of people with avaible records.

**Table 5:** List of variables for systematic review: Demographic, clinical, radiological, pathological, and genetic information extracted.

Items
Patient ID
**Demographic characteristics**
Country
Ethnicity
Sex
Age at the time of study
**Clinical information**
Family history of related phenotype
Age at onset
Disease duration
Initial symptoms
Age of initial diagnosis
Age of clinical diagnosis of NIID
Follow-up time
**Overall symptoms and signs**
Tremor
Cognitive impairment/dementia
Mental disorders (anxiety, depression, irritability, etc)
Abnormal behavior
Muscle fasciculation
Muscle weakness
Sensory disturbance
Autonomic nervous function
Bladder dysfunction
Sexual dysfunction
Constipation
Diarrhea
Vomiting
Hyperhidrosis
Syncope
Miosis
Orthostatic hypotension
Neurological attack
Disturbance of consciousness
Seizure
Encephalitic episode
Stroke-like episode
Other symptoms
Parkinsonism
Rest tremor
Bradykinesia
Rigidity
Ataxia (Gait/limb)
Dystonia
Dyspnea
Dysphagia
Dysarthria
Pyramidal signs
Convulsion
Cough
Headache
Dizziness
Migrane
Vision disorder
Decreased or absent tendon reflex (DTR/ATR)
**Cognitive function test**
MMSE score
MoCA score
Frontal Assessment Battery (FAB) score
**Nerve conduction**
MCV slowing
SCV slowing
CMAP reduction
SNAP reduction
**Cerebrospinal fluid examination**
Cells
Glucose
Protein
**Radiological findings in MRI**
high intensity signal in corticomedullary junction in DWI/ “U-fiber”
Leukoencephalopathy/ white matter hyperintensity
FLAIR high-intensity signals in corpus callosum
Cerebral atrophy/ Ventricular distension
**Pathological findings**
Intra-myonuclear p62 positive
Intra-myonuclear ubiquitin positive
Electron microscopy findings
Pathological changes of nerve and muscle biopsies
**Gene detection results**
Methods of *NOTCH2NLC* gene detection
Number of GGC repeat expansions in *NOTCH2NLC* gene
Results of *FMR1* gene detection

The median AAO was 56.50, 55.00, and 51.50 in tertiles 1, 2, and 3 respectively (*p* trend < 0.001). A significant negative linear correlation was found between GGC repeat expansions and AAO (Y = -0.1344*X + 66.00, *p* < 0.001). For every 10-repeat increase in GGC expansions, symptom onset was observed approximately one year earlier (Fig. 4C). Besides, we observed a rising trend of familial cases as the GGC repeat expansions increased (*p* trend < 0.001), reaching 59.42% in tertile 3 (Table 4). In addition, comparisons between GGC repeat expansions 41-60, 61-100, and 101-250 were carried out and presented in Supplementary Table 4.

Additionally, twenty-two genetically confirmed pairs of parent-offspring with available AAO were identified. The median parental AAO was 60.00 years (IQR: 55.50-65.00). Notably, offspring AAO displayed a statistically significant decrease, with a median of 58.50 years (IQR: 34.75-60.00), indicating a median genetic anticipation of 3.5 years (*p* = 0.039) (Fig. 4D).

## DISCUSSION

To our knowledge, this is the first systematic review of NIID comprehensively exploring the clinical characteristics, neuroimaging features, pathological characteristics, and phenotype-genotype relations in genetically confirmed NIID patients. We provided hitherto undocumented evidence of the high prevalence of DTR/ATR in a large sample size, indicating that the diagnosis of NIID should not be given prior consideration when this neurological sign is absent. Additionally, we summarized the heterogeneity of the four major phenotypes of NIID, based on which a schematic NIID diagnostic framework was proposed. The trend of earlier AAO and higher familial incidences with increasingly larger GGC repeat expansions we first observed in this study may provide important clues for future scientific research, clinical practice, and genetic counseling.

In the following sections, we will discuss our results and summarize the up-to-date information according to the disease course: genes and molecular mechanisms - eosinophilic intranuclear inclusions - neuroimaging features - clinical characteristics - treatments. Furthermore, we have summarized the limitations of this systematic review and anticipations for future research.

### Genes and molecular mechanisms

In 2019, NIID was identified as a short tandem repeat (STR) expansion disorder with GGC repeat expansions in the 5' UTR of the *NOTCH2NLC* gene[13-15, 28]. There are currently more than 40 known STR genes that can cause disease when expanded, and one classic example is Fragile X-associated tremor/ataxia syndrome (FXTAS) [29]. FXTAS is caused by 55-200 CGG repeats in the 5' UTR region of the *FMR1* gene and exhibits potentially molecular similarities with NIID [13, 30]. STR exerts its effects on FXTAS mainly through two mechanisms: RNA gain-of-function and translation of CGG repeats into FMRpolyG, which induces inclusion aggregation and cytotoxicity [31]. Drawing inspiration from the mechanism of FXTAS, researchers found that polyG plays a crucial role in the pathogenesis of NIID [32, 33]. N2NLCpolyG, initiated by an upstream open reading frame (uORF) embedding the GGC repeats, accumulates in p62-positive inclusions. Then, in 2022, Deng et al. unveiled the RNA gain-of-function mechanism underlying NIID pathogenesis [25]. They demonstrated that the *NOTCH2NLC* mRNA forms RNA foci, sequestering RNA-binding proteins (shared with FXTAS) into intranuclear inclusions, thus elucidating the RNA gain-of-function pathway in NIID.

**Figure 5.**
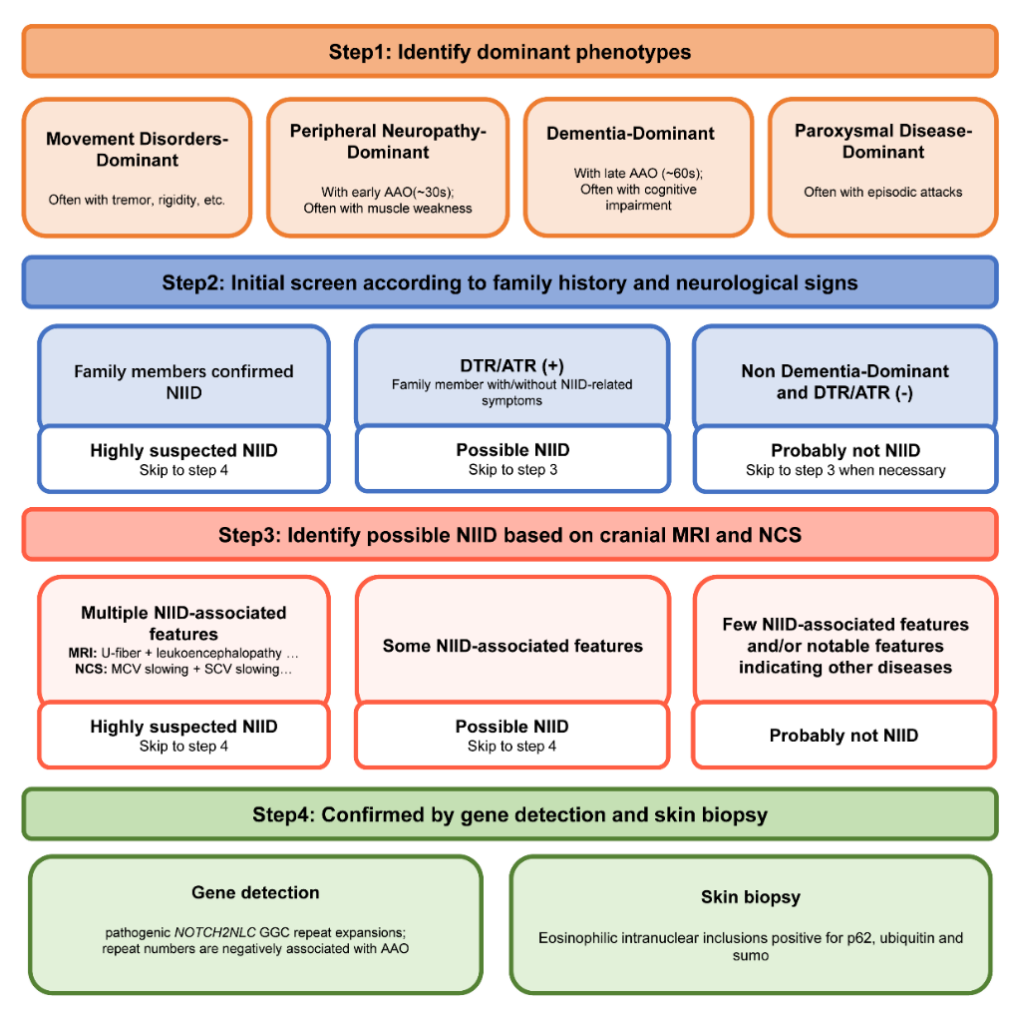
**The schematic framework for NIID diagnosis according to different dominant phenotypes**. Abbreviations: AAO: age at onset; NIID: neuronal intranuclear inclusion disease; DTR/ATR: decreased or absent tendon reflex; MRI: magnetic resonance imaging; MCV: motor nerve conduction velocity; NCS: nerve conduction study; MCV: motor nerve conduction velocity; SCV: sensory nerve conduction velocity.

Current literature indicates that GGC repeat lengths ranging from 7 to approximately 40 are within the physiological range for *NOTCH2NLC* [34]. The upper limit has been proposed to be 300, as GGC repeat expansion in *NOTCH2NLC* exceeding this threshold results in CpG hypermethylation, leading to gene silencing [25]. Consequently, the range of 41 - 300 was adopted in this review. Within this range, we established a significant negative linear correlation between GGC repeat expansions and AAO. Specifically, for every 10-repeat increase in GGC expansions, symptom onset was observed approximately one year earlier. Furthermore, we observed a rising trend of familial cases with the increase of GGC repeat expansions, highlighting the importance of conducting family screenings in clinical practice when the proband exhibits substantial GGC repeat expansions.

### Eosinophilic intranuclear inclusions

In 1980, the disease was initially named “neuronal intranuclear hyaline inclusion disease” due to the widespread presence of intranuclear hyaline inclusions in various types of neurons [3]. These intranuclear inclusions are positive for p62, ubiquitin and sumo [35, 36], observed not only in nerve cells but also glial cells, ganglion cells, Schwann cells, myocardial cells, skeletal muscle cells, renal tubular epithelial cells, and skin sweat gland duct cells [37]. Recent studies utilizing animal antibodies against parts of N2NLCpolyG have unveiled the presence of aberrant proteins within typical ubiquitin and p62-positive intranuclear inclusions in NIID patients, suggesting that the formation of cytoplasmic and intranuclear inclusions is driven by N2NLCpolyG [32, 33]. These intranuclear inclusion have been observed in multiple systems, including the endocrine [38], circulatory [39], gastrointestinal [1, 40], locomotor [41], respiratory [42], urinary [43, 44] and reproductive systems [1, 42]. The extensive involvement of multiple systems and organs may contribute to the high clinical heterogeneity of NIID. However, not all patients in our study exhibited positive pathological test results. Due to dearth of the acquired data, we could not identify common characteristics among these individuals with negative pathological results.

Recently, a study introduced urine cytology as a novel and non-invasive pathological diagnostic technique, even though its positive rate is not as high as of skin biopsy [45]. Future studies utilizing optimized methods of urine cytology are anticipated to validate its diagnostic efficiency.

### Neuroimaging features

The hyperintensity signal in the corticomedullary junction on DWI or subcortical U-fiber, a typical radiological characteristic of NIID, is related to pathological spongiotic changes of brain [7]. Approximately 80% of NIID patients included in this systematic review exhibited U-fiber on DWI, further supporting the potential diagnostic utility of this distinctive sign. However, NIID cases without U-fiber have been reported by different research teams [46-49]. One research following the time course of MRI found that hyperintensities on DWI disappeared after a 5-year follow-up despite disease progression [50]. Besides, though less frequently reported, FXTAS patients can also present a hyperintensity signal in the corticomedullary junction [51]. In summary, U-fiber remains an important diagnostic indicator for probable NIID, particularly in patients dominated by paroxysmal disease and dementia. However, its absence does not conclusively rule out the diagnosis as its presence fluctuates through disease course.

Uniform and symmetrical leukoencephalopathy has been established as one of major MRI presentations in adult-onset NIID [52]. T2/FLAIR hyperintensity signals have been frequently observed in diffuse lesions, including the corpus callosum, internal capsule, thalamus, and basal ganglia [53]. A study confirmed that NIID is the most common cause of adult-onset nonvascular leukoencephalopathies and the second most common cause of adult leukoencephalopathies, following cerebral autosomal dominant arteriopathy with subcortical infarcts and leukoencephalopathy (CADASIL) [54]. Despite diverse inherited or acquired leukoencephalopathies, additional MRI abnormalities and clinical presentations can help narrow the scope of differential diagnosis. U-fiber alone is a remarkable imaging biomarker for differentiating NIID with other leukoencephalopathies, and when DWI is unavailable, incorporating white matter hyperintensity (WMH) in the paravermis or middle cerebellar peduncles (MCPs) into predictive models can achieve high specificity and sensitivity in diagnosing NIID [55]. Additionally, cases of undetermined nonvascular leukoencephalopathy accompanied by symptoms like urinary difficulty, autonomic dysfunction and visual loss are more likely to be caused by NIID [55].

Cerebral atrophy, resulting from neuronal loss and gliosis [52], is also one of the radiological characteristics of NIID. It is particularly prominent in dementia-dominant patients, with its occurrence exceeding 90%. Interestingly, some NIID patients may exhibit only cerebellar atrophy without the presence of high-intensity signals on DWI and T2WI at certain stages of the disease [56]. Ventricular distension, often co-occurring with cerebral atrophy, is also commonly observed in NIID patients, as supported by the results.

### Clinical characteristics

#### Ethnic heterogeneity

Currently reported NIID cases with abnormal GGC repeat expansions in *NOTCH2NLC* are predominantly of Chinese and Japanese descent. NIID patients from other countries in Asia have been reported as well (Korea [57], Malaysia [58], Singapore [59]). However, researchers have noted the difficulty in finding this genetic mutation in European patients with pathologically-confirmed NIID. This suggests that European NIID cases may be driven by a pathophysiological process distinct from Eastern Asian patients, despite both populations showing the common characteristic of abnormal intranuclear inclusions [60].

#### Clinical symptoms and neurological signs

NIID patients can present with a wide range of symptoms. The most common onset symptoms in our study are tremor, cognitive impairment and muscle weakness. This varied onset warrants different attention in clinical settings. Those presenting with cognitive impairment typically experience a later onset, necessitating long-term follow-up for asymptomatic undetermined NIID patients. In cases where muscle weakness is the initial symptom, the proportion of familial cases can be as high as 60%. Therefore, when a patient presents with muscle weakness as the initial symptom, it is advisable to perform familial screening to exclude potential hereditary risks.

Symptoms of autonomic dysfunctions, including bladder dysfunction [61], miosis [62], sexual dysfunction [63], and constipation [64], have been frequently reported after onset. Interestingly, while miosis has been proposed to be a valuable diagnostic indicator for adult-onset NIID [37], in our study, cases with miosis accounted for less than 50%, while the prevalence of another neurological sign, DTR/ATR, was as high as approximately 80%. Tendon reflex is a standard and objective testing method, which can indirectly reflect the degree of injury to the peripheral nerve in neurological clinical practice [65]. DTR/ATR can be attributed to disruptions in the conduction of nerve impulses along the sensorimotor reflex arc caused by either an axonal lesion, conduction block, or myopathy [66]. It is widely thought that NIID is a hereditary demyelinating polyneuropathy characterized by abnormal nerve conduction [67], which may cause an interruption of the sensorimotor reflex arc. This neurological sign can also be seen in other hereditary peripheral neuropathies such as Charcot-Marie-Tooth disease (CMT) [68] and Kennedy disease (KD) [69] in clinical practice. In contrast to CMT and KD, where DTR/ATR tends to be localized in the distal limbs, DTR/ATR in NIID patients is commonly systemic due to its demyelinating polyneuropathy nature [69, 70]. In summary, we recommend DTR/ATR as a notable clinical indicator of NIID that can be utilized in clinical practice.

### Movement disorders

Movement disorders-dominant patients are most common in our study. Its major clinical manifestations including tremor, rigidity, constipation and convulsion. Many patients with this phenotype are initially diagnosed with PD. Recent studies have shed light on the association between *NOTCH2NLC* GGC repeat expansions and PD. In 2020, Ma et al. first reported that patients carrying *NOTCH2NLC* GGC repeat expansions present with typical sporadic PD without any other clinical or imaging features of NIID [71]. Similarly, in 2021, Shi et al. suggested that intermediate-length repeat expansions in *NOTCH2NLC* are potentially associated with PD, and no clinical evidence of NIID was revealed [21]. Taken together, it seems that GGC repeat expansions in *NOTCH2NLC* may be associated with *NOTCH2NLC*-PD instead of NIID-related parkinsonism. However, it is worth noting that undetected NIID cases in prior studies could be due to limited skin biopsy practices. Meanwhile, our study findings demonstrate that only about 40% of movement disorders-dominant patients displayed U-fiber. This indicates that the absence of detectable U-fiber cannot be regarded as definitive evidence to exclude the possibility of NIID in this phenotype.

ET, characterized by bilateral upper extremity action tremor, is one of the most common movement disorders [72]. Similar to PD, there has been a dispute whether ET patients with genetic mutations in *NOTCH2NLC* should be diagnosed with *NOTCH2NLC*-ET or NIID-related ET. In 2020, Sun et al. identified GGC repeat expansions in *NOTCH2NLC* in 11 clinically diagnosed ET families. However, cranial MRI and NCS did not reveal NIID-associated features in the probands. Therefore, it was suggested that those genetically positive families should be clinically diagnosed as ET. However, as we suggested above, undetectable U-fiber in movement disorders-dominant patients does not preclude the possibility of NIID. Moreover, it has been discovered that ET patients could develop new features of NIID during the following-up [59]. In a study by Chen et al., NIID patients who initially lacked any MRI abnormalities were later found to develop U-fiber with disease development [73]. Ultimately, considering cranial MRI features, pathological changes, and genetic evidence, they suggested ET to be an early symptom of NIID.

In summary, when screening for possible NIID in patients with movement disorders, comprehensive evaluations and dynamic observations are crucial. It is important not to dismiss the possibility of NIID solely based on the absence of detectable U-fibers in this phenotype.

### Peripheral neuropathy

In NIID patients, peripheral polyneuropathy is a frequently observed clinical manifestation among various symptoms [48]. Our study adds to the evidence that patients with a dominant peripheral neuropathy phenotype have earlier onset (around 30s) and family history. This might be associated with a larger GGC repeat expansion observed in this phenotype, suggesting its potential role in influencing disease inheritance.

Peripheral neuropathy-dominant patients typically exhibit demyelinating sensorimotor neuropathy with significant MCV and SCV slowing. Intriguingly, even in patients without typical symptoms of peripheral neuropathy, we detected a high frequency of MCV slowing and SCV slowing, indicating the high prevalence of subclinical peripheral neuropathy. Further, NCS revealed that peripheral neuropathy-dominant patients had more prominent CMAP reduction and SNAP reduction than other phenotypes, suggesting more severe axonal degeneration in peripheral neuropathy. These findings highlight the limitations of relying solely on NCS to distinguish peripheral neuropathy-dominant NIID from other inherited neuropathies like CMT that also exhibits slowed velocity and reduced amplitude [74]. Additionally, the presence of U-fiber is relatively uncommon in this phenotype (43.48%), and thus should not be regarded as the sole criteria for excluding NIID.

Oculopharyngodistal myopathy (OPDM), an adult-onset neuromuscular disease characterized by progressive ocular, facial, pharyngeal and distal limb muscle involvement, has also been identified to be associated with GGC repeat expansions in *NOTCH2NLC.* Yu et al. designated OPDM patients with *NOTCH2NLC* mutations as OPDM type 3 (OPDM3) and regarded it as one of the differential diagnoses of peripheral neuropathy-dominant NIID [75]. However, their patients exhibited NIID- associated features, including subclinical peripheral neuropathy, U-fiber, severe leukoencephalopathy, and WMH in periventricular and the corpus callosum. Furthermore, Ogasawara et al. observed the presence of intranuclear inclusions in muscles of initially diagnosed OPDM patients combined with clinical manifestations that partially resembled NIID, indicating that they were more likely of peripheral neuropathy-dominant phenotype rather than *NOTCH2NLC*-OPDM [76].

While underestimated, GGC repeat expansions in *NOTCH2NLC* may significantly contribute to inherited neuropathy. After recognizing the phenotype of peripheral neuropathy, the presence of systemic DTR/ATR and a positive family history can screen potential NIID patients. Further identification requires a combination of NIID-associated features including U-fiber, leukoencephalopathy, MCV slowing and SCV slowing. Ultimately, a definitive diagnosis of NIID hinges on gene detection and skin biopsy.

### Dementia

Dementia refers to progressive decline in intellectual and cognitive function, affecting an individual's daily life and decision-making abilities [77]. Dementia-dominant patients developed symptoms much later (around 60) and have poorer MMSE scores in our study. Intriguingly, less than half of them displayed DTR/ATR, suggesting that this neurological sign is sensitive in certain phenotypes of NIID but dementia. Therefore, the absence of DTR/ATR in patients with non-dementia dominant NIID makes a NIID diagnosis less likely. In this situation, clinicians should consider performing cranial MRI and NCS to evaluate the possibility of NIID when necessary.

In our study, the main clinical manifestations in this phenotype are cognitive impairment, bladder dysfunction and abnormal behaviors. Dementia-dominant patients are often reported to be accompanied with other neurological symptoms [6]. In addition to cognitive problems, they may present with other symptoms including autonomic impairments and paroxysmal symptoms as the disease progresses, indicating a shift towards other phenotypes [23]. This may explain the high prevalence of bladder dysfunction, exceeding 65%, in dementia-dominant patients in our study.

Particularly, the diagnosis of this phenotype should be distinguished from Alzheimer's disease (AD) and frontotemporal dementia (FTD). Tian et al. identified GGC expansion in *NOTCH2NLC* in two AD-affected families [15]. In addition to typical dementia symptoms, affected individuals also displayed other symptoms that are not commonly observed in AD and presented with NIID-associated features on cranial MRI. Skin biopsies further revealed eosinophilic intranuclear inclusions that were positive for p62 and ubiquitin. Considering these findings, a more accurate diagnosis for patients in these two families appears to be dementia-dominant NIID. Intermediate-length GGC repeat expansion in *NOTCH2NLC* was identified in pathologically confirmed AD patients by Wu et al. [78]. They suggested that P62-positive intranuclear inclusions could co-exist with AD neuropathologic changes. However, the mechanism by which the same mutation leads to two pathological patterns warrants further exploration.

Since the clinical features of dementia-dominant phenotype of NIID and neurodegenerative dementia were overlapped, Jiao et al. detected GGC expansion in *NOTCH2NLC* in a cohort of patients with neurodegenerative dementias and found four mutation carriers in FTD patients [79]. Notably, they found four mutation carriers lacked the additional symptoms typically seen in NIID. Additionally, U-fiber was absent in these individuals, but they did exhibit severe leukoencephalopathy. Without dynamic follow-up, it is uncertain whether these patients should be diagnosed with dementia-dominant phenotype of NIID or simply FTD.

Specific diagnostic criteria for this phenotype are currently not well-established. It is crucial to conduct a thorough evaluation and exclude other potential causes of similar symptoms to confirm the diagnosis of the dementia-dominant phenotype. We suggest a four-step diagnostic flow for this phenotype, as illustrated in Figure 5, to guide the diagnostic process.

### Paroxysmal disease

Paroxysmal disease-dominant patients suffer from acute and/or recurrent disease episodes, including consciousness disturbance, stroke-like episodes, encephalitic-like episodes and seizures [80]. The rapid and aggressive nature of this phenotype leads to the shortest disease duration, and the majority of cases (over 85%) are sporadic.

In our study, leukoencephalopathy is the most common neuroimaging feature in this phenotype. It has been reported that NIID patients with encephalopathy commonly exhibit cortical hyperintensities distributed throughout the parietal-occipital lobes, and these lesions do not conform to a specific vascular distribution [55]. Liu et al. found that one-fifth of genetically unsolved nonvascular leukoencephalopathies patients were attributed to NIID, supporting NIID as an underdiagnosed and important entity of adult-onset white matter diseases [55]. Despite diverse inherited or acquired leukoencephalopathies, NIID-associated neuroimaging features including U-fiber, WMH in the paravermis, or MCP combined with clinical presentations can help narrow the scope of differential diagnosis.

Encephalitis-like episodes of NIID should be considered in the differential diagnosis of acute disturbance of consciousness. In encephalitis-like episodes, MRI abnormalities revealed focal hypoperfusion during the acute stage of followed by rebound hyperperfusion later on [54]. Further research is needed to demonstrate whether this feature is specific to this phenotype. Similar to the encephalopathy of NIID, cortical swelling, and hyperperfusion can also present in mitochondrial encephalomyopathy, lactic acidosis, and stroke-like (MELAS) syndrome [81], adding difficulty to the differential diagnosis between MELAS-like episodes in NIID and MELAS syndrome. However, MELAS syndrome patients are often maternally inherited and onset at an early age [82], which is distinct from paroxysmal disease-dominant patients. Meanwhile, NCS of MELAS patients typically show an axonal dominant neuropathy [83], but in NIID patients, demyelination is more frequent than axonal damage.

### A framework of general diagnostic flow for NIID

Based on the extracted available data and the diagnostic value of DTR/ATR, cranial MRI, NCS, skin biopsy, and gene detection, we formulate a framework of general diagnostic flow for NIID (Fig. 5), which aims to provide guidance for future studies and assist NIID diagnosis in clinical practice.

Movement disorders, peripheral neuropathy, dementia and paroxysmal disease are four dominant phenotypes of NIID. In the first step, meticulous history-taking, including initial and present manifestations, AAO, and additional physical examinations, can provide valuable clues for identifying dominant phenotypes.

Secondly, a crucial step in the diagnostic process involves assessing the family history of NIID and determining the presence of DTR/ATR. For patients with a family history, prioritizing the detection of *NOTCH2NLC* gene mutations is essential, given the significant increase in GGC repeat expansions observed in familial cases. According to a previous study [33], we hypothesized that the prolonged GGC repeats harbored by familial cases may increase the aggregation of N2NLCpolyG, leading to more severe impairment of nuclear lamina and nucleocytoplasmic transport, thereby resulting in more serious disease conditions in familial patients. Additionally, consistent with the findings of Tian. et al. [23], our included pedigrees exhibited genetic anticipation, indicating that the severity of NIID may increase in the process of inheritance from parent to offspring [84]. Regarding the presence of DTR/ATR, it has been recognized as a crucial clinical clue suggesting further examinations [27]. Our literature review and data collection revealed that DTR/ATR was present in approximately 80%-90% of NIID patients, except for those with a dementia-dominant phenotype. Therefore, DTR/ATR assessment emerges as a valuable screening tool, particularly for those suspected NIID patients dominated with movement disorders, peripheral neuropathy, or paroxysmal disease.

Thirdly, cranial MRI and NCS play crucial roles as auxiliary examinations in the diagnostic process of NIID. NIID-associated neuroimaging features, including U-fiber [6, 85], leukoencephalopathy [79], WMH in the corpus callosum [86], cerebral atrophy and ventricular distension [52], hold suggestive value of NIID diagnosis. As demonstrated by our study, NCS emerges as the preferred diagnostic approach in patients presenting with peripheral neuropathy. Even in cases without symptoms of muscle weakness and sensory disturbance, NCS still proves valuable in indicating subclinical neuropathy. Furthermore, in patients with negative U-fiber or leukoencephalopathy in MRI, the frequencies of MCV slowing remained relatively high (81.81% and 81.48%, respectively).

In the final step, suspected NIID patients should undergo gene detection and/or skin biopsy in a bid to confirm the final diagnosis. Taking into account the insights derived from previous literature and the findings in this review, along with considerations of cost, invasiveness, and diagnostic accuracy, we believe that this framework, while not yet validated, may offer valuable references and insights for clinical practice and future research.

### Treatment

The treatment of NIID remains challenging as there is currently no recommended effective specific therapy for NIID. Symptomatic treatments, such as anti-Parkinson's disease therapy [21], anti-dizziness therapy [87], and anti-antipsychotic therapy [88], may provide relief for certain symptoms and improve the prognosis for NIID patients. However, these treatments often fail to achieve complete and sustained symptom resolution.

Intriguingly, hormone therapy has demonstrated potential in ameliorating specific symptoms of NIID patients and, in some cases, even slowing the progression of the disease to some extent [89]. Notably, the administration of steroid pulse therapy and maintenance of steroid therapy has been observed to alleviate consciousness disturbance in a case with parkinsonism [90]. Nevertheless, it is essential to acknowledge that hormone treatment could not consistently result in the complete resolution of all abnormalities in lesions or achieve sustained clinical recovery over the long term [81].

Several potential therapies via targeting at the DNA, RNA, and protein levels have been proposed [91]. At the DNA level, gene editing techniques, exemplified by CRISPR/Cas9 technology, have shown promise in excising the STR expansions to repair DNA. This approach has been proven effective in eliminating toxic CGG repeats in the *FMR1* gene [91-93]. Regarding intervention at the RNA level, therapy involving antisense oligonucleotides diseases holds potential. [91, 94].

Idebenone, a CoQ10 analogue to counteract the oxidative stress induced vascular changes in dementia, has been tested for multiple neurological diseases [95]. While it has been applied to NIID patients in some centers, its therapeutic effect remains uncertain, and no related research has been reported.

In summary, despite various treatment options for NIID, such as symptomatic therapies, hormone therapies, and molecular biology therapies, their overall efficacy remains limited. Advances in understanding the underlying pathogenic mechanisms hold promise for developing more effective and targeted therapeutic strategies.

### Limitations

Firstly, inherent selection biases and information biases are unavoidable, leading to some deviations between our conclusions and actual clinical practice. Publication bias poses a potential limitation, as statistically significant studies are more likely to be published than those with negative findings. Additionally, articles published in some major languages such as Japanese or Chinese were not included. Given that NIID cases are rarely reported outside of East Asia, our collection is limited in terms of global generalizability. Furthermore, due to the lack of unified reporting standards and the clinical heterogeneity of NIID, the collection of clinical information lacks systematic processes and consistent standards across different centers, resulting in inevitable information bias. For example, insufficient data on less reported variables like WMH in the paravermis. Therefore, to mitigate these biases, we proposed a list of extracted variables (Table 5) that could serve as a standard for NIID research, promoting consistency and comparability across studies. Secondly, we acknowledge statistical bias. Some data from observational studies with large sample size were unavailable, potentially leading to inconsistent frequency estimates of certain symptoms compared to those reported in the literature. Besides, the variability in sequencing techniques used by different centers could introduce bias into our overall findings regarding GGC repeat expansions. Thirdly, existing research suggests that NIID phenotypes can transition over time, with new symptoms emerging [23], and the neuroimaging features can evolve as the disease progresses [50]. As a systematic review, we were unable to delve into the longitudinal course of these changes or their underlying mechanisms. More longitudinal cohort studies are needed to clarify NIID progression and its causes. Lastly, certain conclusions from our study necessitate validation through future research efforts. Given the existing data limitations, we could not quantitatively assess the diagnostic value of indicators like DTR/ATR for NIID. Furthermore, the schematic framework we proposed lacks clinical validation and should undergo further quantification and optimization in the future to ensure its effectiveness in clinical practice.

While we acknowledge the potential limitations of genotype-phenotype characterization through systematic review, we believe this method to be robust when reviewing such rare diseases, where aggregating data from multiple small case reports and series can reveal meaningful insights. This method should be used to continually update our clinical knowledge as new cases emerge and more data becomes available for analysis.

### Future anticipations

Currently, there is a notable scarcity of animal models and foundational research on NIID. There is a pressing need for more studies to unravel the mechanisms underlying the onset and progression of NIID and the fundamental mechanisms governing its characteristic manifestations. Additionally, there is currently no effective treatment for NIID. Clinical management mainly relies on symptomatic approaches. Therefore, it is imperative to intensify efforts in targeting drug development and expedite their clinical trials.

Due to the relative rarity and high heterogeneity of NIID, clinical diagnosis is typically challenging, and the absence of comprehensive, scientifically grounded, universally applicable diagnostic criteria compound these difficulties. Further research is essential to simplify screening and identify more valuable diagnostic features. A quantified and optimized NIID diagnostic workflow is a crucial next step.

Lastly, we expect this study systematically reviewing the clinical characteristics, neuroimaging features, pathological characteristics and phenotype-genotype relations could deepen and expand understandings of NIID, contributing to the standardization of clinical information collection and the identification of NIID patients in clinical practice.

## CONCLUSIONS

We have summarized the most comprehensive and up-to-date genotypic and phenotypic information on NIID since the identification of the causative gene *NOTCH2NLC*. We are the first to confirm the increasing trend of familial patients and earlier onset with the increase of GGC repeat expansions. Tremor, cognitive impairment and muscle weakness are the three most prevalent initial symptoms. DTR/ATR emerges as a notable clinical indicator of NIID that can be utilized in routine clinical practice. Among the identified phenotypes, paroxysmal disease-dominant patients exhibit earlier onset and a higher familial incidence, possibly linked to larger GGC repeat expansions. Our study contributes a novel diagnostic framework for NIID, offering valuable insights to enhance clinical practice.

### Ethics approval

Not applicable. This study was a secondary analysis, and all patient data were derived from published studies.

## Supplementary Materials

The Supplementary data can be found online at: www.aginganddisease.org/EN/10.14336/AD.2024.0131.


