# Supplementary material for: Neuronal Intranuclear Inclusion Disease with NOTCH2NLC GGC Repeat Expansion: A Systematic Review and Challenges of Phenotypic Characterization
Source: Aging Dis. 2024 Jan 31;16(1):578–97. doi: 10.14336/AD.2024.0131-1 (PMC11745434; doi:10.14336/AD.2024.0131-1)
Supplement: Supplementary file 1 [file AD-16-1-578-s.pdf]

SUPPLEMENTARY DATA

Neuronal Intranuclear Inclusion Disease with *NOTCH2NLC* GGC Repeat Expansion: A Systematic Review and Challenges of Phenotypic Characterization

Tian Zeng, Yiqun Chen, Honghao Huang, Shengqi Li, Jiaqi Huang, Haobo Xie, Shenyi Lin, Siyao Chen, Guangyong Chen, Dehao Yang

© 2024. Zeng T et al. Published online at http://www.aginganddisease.org/EN/10.14336/AD.2024.0131-1

SUPPLEMENTARY DATA

Supplementary Table 1. Characteristics and quality assessment of 85 included researches meeting the criteria for systematic review.

Ref	First author	Year	Country/Region	Included sample size	Range of GGC repeat expansions	Quality assessment						
						Total scores	Sequence analysis	Imaging examination	Pathological test	Nerve conduction	Demographic data	Clinical assessment
1 [1]	Yun Tian	2019	China	45	66-517	6	1	1	1	1	1	1
2 [2]	Bin Jiao	2020	China	4	76-133	5	2	1	0	0	1	1
3 [3]	Yanchun Yuan	2020	China	7	44-143	7	2	1	1	1	1	1
4 [4]	Xun Zhou	2022	China	50	60-250	5	2	1	0	0	1	1
5 [5]	Jianwen Deng	2019	China	15	Not available	4	0	1	1	0	1	1
6 [6]	Jianwen Deng	2021	China	4	41-300	7	2	1	1	1	1	1
7 [7]	Jiaxi Yu	2021	China	3	68-306	7	2	1	1	1	1	1
8 [8]	Danhua Zhao	2021	China	1	81	7	2	1	1	1	1	1
9 [9]	Hui Wang	2021	China	5	126-206	7	2	1	1	1	1	1
10 [10]	Jiaxi Yu	2021	China	5	128-198	7	2	1	1	1	1	1
11 [11]	Zi-Yi Wang	2022	China	1	105	7	2	1	1	1	1	1
12 [12]	Xuejun Guo	2022	China	1	108	6	2	1	1	0	1	1
13 [13]	Daojun Hong	2022	China	28	82-143	7	2	1	1	1	1	1
14 [14]	Fan Li	2022	China	2	Not available	4	0	1	1	0	1	1
15 [15]	Wei Wu	2022	China	1	43	6	2	1	1	0	1	1
16 [16]	Huiting Liang	2020	China	4	98-123	7	2	1	1	1	1	1
17 [17]	Pu Fang	2020	China	5	101-266	6	2	1	1	0	1	1
18 [18]	Chengsi Wu	2022	China	2	≥100	6	1	1	1	1	1	1
19 [19]	Yiyi Zhou	2022	China	10	96-158	6	1	1	1	1	1	1
20 [20]	Shugang Zhang	2020	China	4	≥99	7	2	1	1	1	1	1
21 [21]	Mingming Li	2020	China	1	96	7	2	1	1	1	1	1
22 [22]	Hao Chen	2020	China	51	>60	5	1	1	1	0	1	1
23 [23]	Hao Chen	2020	China	16	Not available	3	0	1	1	0	0	1
24 [24]	Ying Huang	2021	China	1	102	7	2	1	1	1	1	1
25 [25]	Wei-Ping Deng	2021	China	1	115	7	2	1	1	1	1	1
26 [26]	Yuwen Cao	2021	China	19	64-180	6	2	1	1	0	1	1
27 [27]	Yuwen Cao	2022	China	25	≥60	6	2	1	1	0	1	1
28 [28]	Yuwen Cao	2022	China	1	100	3	2	1	0	0	0	0
29 [29]	Yaping Yan	2021	China	3	83-102	4	1	1	0	0	1	1
30 [30]	Hui Dong	2020	China	1	88	6	2	1	1	0	1	1
31 [31]	Guang Ji	2022	China	1	121	7	2	1	1	1	1	1
32 [32]	Jiao-Jiao Guo	2020	China	1	72	6	2	1	1	0	1	1
33 [33]	Gao-Jia Zhang	2022	China	1	97	6	2	1	1	0	1	1
34 [34]	Chang-He Shi	2020	China	12	41-52	6	2	1	1	0	1	1

© 2024. Zeng T et al. Published online at http://www.aginganddisease.org/EN/10.14336/AD.2024.0131-1

SUPPLEMENTARY DATA

35 [35]	Yu Fan	2021	China	1	50	5	2	0	1	0	1	1
36 [36]	Jie Pang	2021	China	5	66-142	5	2	1	1	0	0	1
37 [37]	Yutao Liu	2022	China	2	66-70	7	2	1	1	1	1	1
38 [38]	Yun-Chao Wang	2022	China	9	41-98	5	2	1	0	0	1	1
39 [39]	Jiadi Li	2022	China	1	93	3	2	0	0	0	1	0
40 [40]	Dehao Yang	2022	China	14	≥95	7	2	1	1	1	1	1
41 [41]	Dehao Yang	2022	China	3	90-124	7	2	1	1	1	1	1
42 [42]	Hongfen Wang	2022	China	1	107	7	2	1	1	1	1	1
43 [43]	Fei Xie	2022	China	1	118	5	2	1	0	0	1	1
44 [44]	Chang Liu	2022	China	6	≥84	6	2	1	1	1	0	1
45 [45]	Wei Zhang	2022	China	3	>60	7	2	1	1	1	1	1
46 [46]	Qian Zhou	2022	China	1	>66	6	1	1	1	1	1	1
47 [47]	Ning Su	2022	China	1	101	6	2	1	1	0	1	1
48 [48]	Jibao Wu	2022	China	1	Not available	4	0	1	1	0	1	1
49 [49]	Chujun Wu	2022	China	39	87-159	5	2	1	0	0	1	1
50 [50]	Xue Gao	2022	China	1	Not available	3	0	1	0	0	1	1
51 [51]	Bo Zhao	2022	China	2	131-148	7	2	1	1	1	1	1
52 [52]	Yue Lou	2022	China	1	106	7	2	1	1	1	1	1
53 [53]	Jun Sone	2019	Japan	61	71-183	7	2	1	1	1	1	1
54 [54]	Keisuke Mizutani	2022	Japan	1	Not available	4	0	1	0	1	1	1
55 [55]	Hiroyuki Ishiura	2019	Japan	32	90-180	5	2	1	0	0	1	1
56 [56]	Natsuko Nakamura	2020	Japan	2	113-116	5	2	1	0	0	1	1
57 [57]	Hiroyuki Ishiura	2020	Japan	2	137-140	4	1	1	1	0	0	1
58 [58]	Shun Okamura	2020	Japan	1	Not available	5	0	1	1	1	1	1
59 [59]	Takaaki Hayashi	2020	Japan	1	94	7	2	1	1	1	1	1
60 [60]	Kisaki Tachi	2021	Japan	1	Not available	5	0	1	1	1	1	1
61 [61]	Masaki Okubo	2019	Japan	12	89-143	6	2	1	0	1	1	1
62 [62]	Masashi Ogasawara	2020	Japan	7	116-674	7	2	1	1	1	1	1
63 [63]	Masashi Ogasawara	2022	Japan	10	Not available	3	0	1	1	0	0	1
64 [64]	Masashi Ogasawara	2022	Japan	1	Not available	3	0	1	1	0	0	1
65 [65]	Mai Kikumoto	2021	Japan	1	Not available	4	0	1	1	0	1	1
66 [66]	Hiromi Fukuda	2021	Japan	5	93-650	7	2	1	1	1	1	1
67 [67]	Shinichi Kameyama	2022	Japan	3	76-112	7	2	1	1	1	1	1
68 [68]	Yosuke Miyamoto	2022	Japan	1	108	7	2	1	1	1	1	1
69 [69]	Tomone Taneda	2022	Japan	1	89	6	2	1	1	0	1	1
70 [70]	Ryo Tokimura	2022	Japan	1	Not available	4	0	1	1	0	1	1
71 [71]	Jun-Hui Yuan	2022	Japan	1	113	4	2	1	0	0	0	1
72 [72]	Atsuhiko Sugiyama	2022	Japan	1	Not available	3	0	1	0	0	1	1
73 [73]	Azusa Orihara	2022	Japan	1	Not available	4	0	1	1	0	1	1

© 2024. Zeng T et al. Published online at http://www.aginganddisease.org/EN/10.14336/AD.2024.0131-1

74 [74]	Kazuhiro Fukushima	2022	Japan	1	130	6	2	1	1	0	1	1
75 [75]	Masanori Kurihara	2022	Japan	3	Not available	4	0	1	1	0	1	1
76 [76]	Taku Homma	2022	Japan	1	Not available	4	0	1	1	0	1	1
77 [77]	Na-Yeon Jung	2022	Korea	2	107-149	6	2	1	1	0	1	1
78 [78]	Zhiyong Chen	2020	Singapore	12	92-138	6	2	1	1	0	1	1
79 [79]	Adeline S. L. Ng	2020	Singapore	4	80-107	6	2	1	1	0	1	1
80 [80]	Dongrui Ma	2020	Singapore	13	41-130	4	2	1	0	0	0	1
81 [81]	Yi-Chu Liao	2021	Taiwan	9	80-104	7	2	1	1	1	1	1
82 [82]	Yun Hsia	2021	Taiwan	1	Not available	4	0	1	1	0	1	1
83 [83]	Yi Hong Liu	2022	Taiwan	17	73-323	6	2	1	1	0	1	1
84 [84]	Zhongbo Chen	2020	UK	1	58	6	2	1	1	0	1	1
85 [85]	Wai Yan Yau	2021	UK	2	90-118	7	2	1	1	1	1	1

Notes: Domain 1. sequence analysis: 2 - reporting precise number of repeat expansions; 1- reporting unspecified number of repeat expansions (e.g. only reporting a range); 0 - not reporting the number of GGC repeat expansions.
Domain 2. imaging examination: 1 - reporting the results of imaging examination; 0 - without relevant data.
Domain 3. pathological test: 1 - reporting the results of pathological tests; 0 - without relevant data.
Domain 4. nerve conduction: 1 - reporting the results of nerve conduction study; 0 - without relevant data.
Domain 5. demographic data: 1 - reporting age at onset, sex and age; 0 - without relevant data.
Domain 6. clinical assessment: 1 - reporting clinical presentations or signs; 0 - without relevant data.

Supplementary Table 2. Detailed information of index cases with GGC repeat expansion > 300.

ID	AAO	Familial	Sex	GGC repeats	MRI tests	Pathological tests	Initial symptoms	Reference
**F1-1**	-	+	Male	371	Negative	Negative	-	[6]
**F2-1**	-	+	Male	709	Negative	Negative	-	[6]
**F2-IV-5 (Patient 2)**	26	+	Male	306	Negative	Positive	Hand tremor	[7]
**Patient 2**	27	-	Female	674	Negative	Positive	Muscle weakness	[62]
**F1_Fa**	-	+	Male	522	NA	NA	-	[66]
**F2_Fa**	-	+	Male	390	NA	NA	-	[66]
**F3_Fa**	-	+	Male	528	NA	Negative	-	[66]
**F4_Fa**	-	+	Male	650	NA	Negative	-	[66]
**P231**	NA	-	NA	323	NA	NA	NA	[83]
**Abbreviations:** AAO: Age at onset; NA: Not available.								

Supplementary Table 3. Detailed information of index cases with negative pathological tests.

ID	AAO	Familial	Sex	GGC repeats	MRI tests	Pathological tests	Initial symptoms	Reference
**P20 (T5716)**	34	+	Female	>180	Negative	Negative	Muscle weakness	[26]
	61	NA	Female	50	NA	Negative	Tremor and muscle rigidity	[35]
**4**	52	-	Male	138	Positive	Negative	Urinary symptoms	[78]
**5**	63	-	Female	121	Positive	Negative	Dementia	[78]
**6**	50	-	Male	138	Positive	Negative	Urinary symptoms	[78]
**9**	60	+	Male	128	Positive	Negative	Gait ataxia	[78]
**Abbreviations:** AAO: Age at onset; NA: Not available.								

© 2024. Zeng T et al. Published online at http://www.aginganddisease.org/EN/10.14336/AD.2024.0131-1

SUPPLEMENTARY DATA

Supplementary Table 4. The relationship between phenotype and genotype.

	GGC repeats 41-60 (n = 35)	GGC repeats 61-100 (n = 101)	GGC repeats 101-250 (n = 234)	*P trend*
**AAO, median (IQR)**	60.50 (51.00-62.00) [22]	56.00 (48.50-64.00) [84]	53.00 (40.00-60.00) [202]	$p = 0.001$
**AAR, median (IQR)**	62.25 (61.00-65.00) [22]	65.00 (56.00-69.00) [82]	62.00 (51.00-67.00) [160]	$p = 0.071$
**Duration, median (IQR)**	2.00 (0.00-6.25) [21]	6.00 (1.50-10.00) [72]	9.00 (4.00-13.25) [145]	$p < 0.001$
**Familial, No. (%)**	0 (0/31)	33.33 (25/75)	51.67 (62/120)	$p < 0.001$
**Male, No. (%)**	52.17 (12/23)	40.66 (37/91)	46.88 (105/224)	$p = 0.801$
**Tremor, No. (%)**	82.61 (19/23)	57.89 (44/76)	64.86 (120/185)	$p = 0.510$
**Cognitive impairment**	34.48 (10/29)	65.00 (52/80)	65.43 (123/188)	$p = 0.312$
**Muscle weakness**	76.47 (13/17)	39.19 (29/74)	35.37 (58/164)	$p = 0.008$
**Consciousness Disturbance**	0 (0/3)	34.88 (15/43)	22.86 (24/105)	$p = 0.397$
**DTR/ATR**	0 (0/3)	82.61 (19/23)	80.28 (57/71)	$p = 0.078$
**MRI Tests**				
U-fiber	0 (0/11)	79.63 (43/54)	72.63 (69/95)	$p = 0.005$
Leukoencephalopathy	38.89 (7/18)	93.75 (30/32)	89.62 (95/106)	$p < 0.001$
Cerebral atrophy	5.56 (1/18)	78.57 (22/28)	78.13 (25/32)	$p < 0.001$
**Nerve conductions**				
MCV slowing	50.00 (1/2)	71.43 (15/21)	82.81 (53/64)	$p = 0.135$
SCV slowing	50.00 (1/2)	76.00 (19/25)	81.25 (52/64)	$p = 0.326$
**Pathological Tests**	80.00 (4/5)	100.00 (60/60)	96.67 (116/120)	$p = 0.927$
**Abbreviations**: AAO: age at onset; AAR: age at research; DTR/ATR: decreased or absence of tendon reflex				

Supplementary Table 5. Summary of the data source of clinical manifestations.

Clinical manifestations	Literatures reporting positive symptoms	Literatures reporting negative symptoms
Tremor	Ref 3, Ref 4, Ref 5, Ref 7, Ref 8, Ref 11, Ref 12, Ref 13, Ref 14, Ref 16, Ref 17, Ref 18, Ref 19, Ref 20, Ref 23, Ref 25, Ref 26, Ref 27, Ref 29, Ref 40, Ref 42, Ref 45, Ref 49, Ref 51, Ref 55, Ref 56, Ref 61, Ref 62, Ref 69, Ref 70, Ref 71, Ref 74, Ref 79, Ref 81	Ref 3, Ref 5, Ref 6, Ref 10, Ref 13, Ref 14, Ref 15, Ref 16, Ref 17, Ref 19, Ref 20, Ref 26, Ref 27, Ref 40, Ref 41, Ref 49, Ref 51, Ref 61, Ref 62
Bradykinesia	Ref 1, Ref 19, Ref 34, Ref 38, Ref 50, Ref 79, Ref 80, Ref 85	Ref 1, Ref 3, Ref 4, Ref 15, Ref 19, Ref 51, Ref 57, Ref 69, Ref 85
Rigidity	Ref 1, Ref 5, Ref 34, Ref 35, Ref 38, Ref 42, Ref 52, Ref 55, Ref 67, Ref 80	Ref 1, Ref 3, Ref 5, Ref 6, Ref 9, Ref 15, Ref 34, Ref 51, Ref 52, Ref 60, Ref 67, Ref 69, Ref 85
Ataxia	Ref 1, Ref 21, Ref 25, Ref 27, Ref 31, Ref 32, Ref 49, Ref 55, Ref 57, Ref 60, Ref 62, Ref 69, Ref 70, Ref 72, Ref 74, Ref 76	Ref 1, Ref 3, Ref 4, Ref 5, Ref 6, Ref 9, Ref 14, Ref 15, Ref 16, Ref 17, Ref 19, Ref 26, Ref 29, Ref 37, Ref 40, Ref 41, Ref 50, Ref 51, Ref 53, Ref 66, Ref 67, Ref 78, Ref 80, Ref 81
Gait	Ref 7, Ref 10, Ref 18, Ref 21, Ref 25, Ref 32, Ref 34, Ref 45, Ref 50, Ref 53, Ref 55, Ref 60, Ref 68, Ref 69, Ref 74, Ref 78, Ref 81, Ref 82	Ref 34, Ref 78
Cognitive impairment	Ref 2, Ref 3, Ref 5, Ref 6, Ref 8, Ref 9, Ref 11, Ref 12, Ref 13, Ref 14, Ref 15, Ref 16, Ref 17, Ref 19, Ref 20, Ref 21, Ref 24, Ref 26, Ref 27, Ref 30, Ref 32, Ref 33, Ref 34, Ref 36, Ref 37, Ref 38, Ref 40, Ref 41, Ref 46, Ref 49, Ref 50, Ref 51, Ref 52, Ref 53, Ref 55, Ref 57, Ref 60, Ref 61, Ref 67, Ref 71, Ref 72, Ref 73, Ref 74, Ref 75, Ref 76, Ref 77, Ref 78, Ref 79, Ref 80, Ref 81, Ref 82, Ref 85	Ref 3, Ref 4, Ref 5, Ref 6, Ref 7, Ref 9, Ref 10, Ref 14, Ref 19, Ref 27, Ref 31, Ref 40, Ref 41, Ref 49, Ref 53, Ref 79, Ref 80, Ref 81, Ref 85
Bladder dysfunction	Ref 1, Ref 3, Ref 5, Ref 8, Ref 9, Ref 11, Ref 14, Ref 16, Ref 17, Ref 19, Ref 20, Ref 26, Ref 27, Ref 30, Ref 42, Ref 43, Ref 45, Ref 52, Ref 53, Ref 54, Ref 55, Ref 57, Ref 61, Ref 77, Ref 78, Ref 79	Ref 1, Ref 5, Ref 6, Ref 14, Ref 16, Ref 19, Ref 26, Ref 40, Ref 51, Ref 53, Ref 61, Ref 62, Ref 66, Ref 78, Ref 79
Sexual dysfunction	Ref 9, Ref 22, Ref 57, Ref 61	Ref 9, Ref 22, Ref 61

© 2024. Zeng T et al. Published online at http://www.aginganddisease.org/EN/10.14336/AD.2024.0131-1

SUPPLEMENTARY DATA

Constipation	Ref 1, Ref 9, Ref 11, Ref 17, Ref 19, Ref 25, Ref 27, Ref 32, Ref 34, Ref 36, Ref 41, Ref 53, Ref 55, Ref 61, Ref 78	Ref 9, Ref 17, Ref 19, Ref 27, Ref 34, Ref 36, Ref 53, Ref 55, Ref 61, Ref 78
Diarrhea	Ref 9, Ref 36, Ref 65	Ref 9, Ref 36
Hyperhidrosis	Ref 9, Ref 32, Ref 36, Ref 71	Ref 9, Ref 36
Orthostatic hypotension	Ref 9, Ref 17, Ref 27, Ref 36, Ref 53, Ref 71, Ref 78	Ref 9, Ref 17, Ref 27, Ref 34, Ref 36, Ref 42, Ref 53, Ref 78, Ref 85
Miosis	Ref 1, Ref 5, Ref 11, Ref 12, Ref 16, Ref 19, Ref 22, Ref 26, Ref 27, Ref 30, Ref 36, Ref 40, Ref 46, Ref 53, Ref 55, Ref 61, Ref 62, Ref 66, Ref 67, Ref 68, Ref 70	Ref 1, Ref 3, Ref 5, Ref 6, Ref 8, Ref 16, Ref 19, Ref 22, Ref 26, Ref 27, Ref 36, Ref 40, Ref 51, Ref 53, Ref 60, Ref 61, Ref 62, Ref 67
Vomit	Ref 6, Ref 8, Ref 16, Ref 19, Ref 21, Ref 26, Ref 27, Ref 32, Ref 37, Ref 43, Ref 45, Ref 46, Ref 53, Ref 54, Ref 55, Ref 58, Ref 61, Ref 65, Ref 68, Ref 71, Ref 73, Ref 75, Ref 76, Ref 77, Ref 83, Ref 85	Ref 6, Ref 16, Ref 19, Ref 20, Ref 26, Ref 27, Ref 51, Ref 53, Ref 61, Ref 83, Ref 85
Muscle weakness	Ref 1, Ref 3, Ref 6, Ref 7, Ref 8, Ref 9, Ref 10, Ref 14, Ref 16, Ref 17, Ref 18, Ref 19, Ref 25, Ref 26, Ref 27, Ref 29, Ref 30, Ref 31, Ref 33, Ref 36, Ref 37, Ref 38, Ref 42, Ref 45, Ref 46, Ref 49, Ref 51, Ref 53, Ref 58, Ref 61, Ref 62, Ref 63, Ref 66, Ref 68, Ref 71, Ref 80, Ref 81	Ref 1, Ref 3, Ref 4, Ref 13, Ref 14, Ref 15, Ref 16, Ref 17, Ref 19, Ref 20, Ref 21, Ref 24, Ref 26, Ref 27, Ref 29, Ref 36, Ref 38, Ref 40, Ref 41, Ref 49, Ref 50, Ref 51, Ref 53, Ref 54, Ref 61, Ref 63, Ref 67, Ref 81
Muscle fasciculation	Ref 3, Ref 5, Ref 7, Ref 18, Ref 53, Ref 81	Ref 3, Ref 5, Ref 15, Ref 18, Ref 29, Ref 42, Ref 53, Ref 68, Ref 80
Sensory disturbance	Ref 1, Ref 3, Ref 5, Ref 7, Ref 9, Ref 16, Ref 19, Ref 25, Ref 26, Ref 27, Ref 36, Ref 37, Ref 38, Ref 43, Ref 48, Ref 49, Ref 53, Ref 55, Ref 58, Ref 61, Ref 62, Ref 66, Ref 68, Ref 71, Ref 73, Ref 77, Ref 80, Ref 81, Ref 85	Ref 1, Ref 3, Ref 4, Ref 5, Ref 6, Ref 7, Ref 9, Ref 10, Ref 13, Ref 15, Ref 16, Ref 18, Ref 19, Ref 26, Ref 27, Ref 29, Ref 31, Ref 36, Ref 37, Ref 38, Ref 40, Ref 45, Ref 49, Ref 51, Ref 53, Ref 55, Ref 61, Ref 62, Ref 67, Ref 77, Ref 80, Ref 85
Decreased or absence of tendon reflex	Ref 6, Ref 7, Ref 8, Ref 9, Ref 10, Ref 12, Ref 13, Ref 18, Ref 19, Ref 21, Ref 25, Ref 30, Ref 31, Ref 32, Ref 40, Ref 41, Ref 42, Ref 43, Ref 45, Ref 46, Ref 51, Ref 55, Ref 58, Ref 60, Ref 61, Ref 62, Ref 70, Ref 73, Ref 79, Ref 81, Ref 85	Ref 3, Ref 11, Ref 13, Ref 15, Ref 19, Ref 40, Ref 52, Ref 61
Consciousness disturbance	Ref 1, Ref 4, Ref 6, Ref 8, Ref 11, Ref 16, Ref 21, Ref 26, Ref 27, Ref 36, Ref 37, Ref 49, Ref 52, Ref 53, Ref 54, Ref 55, Ref 60, Ref 65, Ref 66, Ref 67, Ref 68, Ref 73, Ref 75, Ref 77	Ref 1, Ref 3, Ref 4, Ref 6, Ref 9, Ref 20, Ref 26, Ref 27, Ref 29, Ref 36, Ref 37, Ref 40, Ref 43, Ref 49, Ref 51, Ref 53, Ref 55, Ref 61, Ref 62, Ref 67, Ref 85
Seizure	Ref 6, Ref 11, Ref 19, Ref 26, Ref 27, Ref 30, Ref 49, Ref 52, Ref 60, Ref 66, Ref 78, Ref 83, Ref 85	Ref 4, Ref 6, Ref 19, Ref 26, Ref 27, Ref 42, Ref 49, Ref 54, Ref 55, Ref 61, Ref 62, Ref 83, Ref 85
Stroke-like episode	Ref 1, Ref 24, Ref 26, Ref 27, Ref 43, Ref 46, Ref 49, Ref 51, Ref 55, Ref 60, Ref 61, Ref 65, Ref 77	Ref 1, Ref 3, Ref 15, Ref 16, Ref 26, Ref 27, Ref 40, Ref 49, Ref 51, Ref 52, Ref 67, Ref 73, Ref 77, Ref 85
Encephalitic episode	Ref 1, Ref 12, Ref 21, Ref 24, Ref 26, Ref 30, Ref 32, Ref 49, Ref 53, Ref 54, Ref 61, Ref 66, Ref 67, Ref 68, Ref 73, Ref 75, Ref 76, Ref 78, Ref 83, Ref 84, Ref 85	Ref 1, Ref 3, Ref 6, Ref 9, Ref 26, Ref 27, Ref 40, Ref 49, Ref 53, Ref 61, Ref 62, Ref 67, Ref 75, Ref 78, Ref 85
Dystonia	Ref 8, Ref 9, Ref 11, Ref 19, Ref 25, Ref 26, Ref 33, Ref 46, Ref 53	Ref 21, Ref 26, Ref 32, Ref 40, Ref 57, Ref 74
Dysphagia	Ref 3, Ref 9, Ref 10, Ref 20, Ref 25, Ref 27, Ref 31, Ref 53, Ref 55, Ref 62, Ref 63, Ref 66, Ref 68, Ref 71	Ref 3, Ref 9, Ref 42, Ref 45, Ref 53, Ref 60, Ref 62, Ref 63
Dysarthria	Ref 3, Ref 5, Ref 6, Ref 10, Ref 16, Ref 20, Ref 21, Ref 24, Ref 25, Ref 27, Ref 30, Ref 31, Ref 32, Ref 37, Ref 38, Ref 42, Ref 45, Ref 46, Ref 53, Ref 57, Ref 62, Ref 65, Ref 71, Ref 76, Ref 81, Ref 83, Ref 85	Ref 3, Ref 5, Ref 16, Ref 27, Ref 45, Ref 60, Ref 69, Ref 83, Ref 85
Pyramidal signs	Ref 3, Ref 8, Ref 11, Ref 17, Ref 19, Ref 51	Ref 9, Ref 17, Ref 19, Ref 32, Ref 40, Ref 42, Ref 60, Ref 68, Ref 69, Ref 73
Convulsion	Ref 3, Ref 5, Ref 6, Ref 30, Ref 32, Ref 34, Ref 53, Ref 81	Ref 5, Ref 6, Ref 9, Ref 20, Ref 25, Ref 34, Ref 53, Ref 60

© 2024. Zeng T et al. Published online at http://www.aginganddisease.org/EN/10.14336/AD.2024.0131-1

Cough	Ref 6, Ref 9, Ref 18, Ref 19, Ref 20, Ref 27, Ref 37, Ref 52	Ref 6, Ref 9, Ref 19, Ref 27, Ref 37
Headache	Ref 6, Ref 8, Ref 16, Ref 20, Ref 26, Ref 27, Ref 30, Ref 33, Ref 36, Ref 37, Ref 38, Ref 43, Ref 46, Ref 47, Ref 49, Ref 51, Ref 55, Ref 65, Ref 66, Ref 73, Ref 83, Ref 84, Ref 85	Ref 25, Ref 26, Ref 27, Ref 36, Ref 49, Ref 51, Ref 83, Ref 85
Dizziness	Ref 8, Ref 9, Ref 13, Ref 20, Ref 21, Ref 26, Ref 34, Ref 38, Ref 43, Ref 49, Ref 55, Ref 61, Ref 70, Ref 75, Ref 77, Ref 85	Ref 25, Ref 26, Ref 34, Ref 43, Ref 51, Ref 77, Ref 85
Vision	Ref 5, Ref 6, Ref 10, Ref 13, Ref 16, Ref 17, Ref 19, Ref 20, Ref 22, Ref 26, Ref 27, Ref 37, Ref 43, Ref 44, Ref 46, Ref 47, Ref 49, Ref 51, Ref 53, Ref 55, Ref 59, Ref 62, Ref 70, Ref 76, Ref 77, Ref 82	Ref 5, Ref 6, Ref 13, Ref 16, Ref 17, Ref 19, Ref 22, Ref 26, Ref 27, Ref 49, Ref 51, Ref 55, Ref 62, Ref 77
Hypertension	Ref 1, Ref 5, Ref 9, Ref 14, Ref 24, Ref 30, Ref 32, Ref 38, Ref 45, Ref 51, Ref 53, Ref 55, Ref 57	Ref 5, Ref 11, Ref 14, Ref 25, Ref 38, Ref 52, Ref 55
Abnormal behavior	Ref 1, Ref 2, Ref 3, Ref 5, Ref 16, Ref 26, Ref 30, Ref 48, Ref 52, Ref 53, Ref 60, Ref 61, Ref 75	Ref 1, Ref 2, Ref 3, Ref 5, Ref 9, Ref 16, Ref 26, Ref 40, Ref 53, Ref 67
Mental disorders	Ref 2, Ref 8, Ref 19, Ref 21, Ref 25, Ref 27, Ref 30, Ref 32, Ref 38, Ref 42, Ref 46, Ref 49, Ref 51, Ref 55, Ref 60, Ref 61, Ref 77, Ref 83	Ref 2, Ref 19, Ref 24, Ref 49, Ref 83

SUPPLEMENTARY RESULTS

Patients harboring expansion repeats > 300

Out of a total of 414 cases with available specific GGC repeats records, 9 of them harbored expansion repeats > 300, which were considered nonpathogenic by Deng et al. [6]. Therefore, these nine cases were excluded from the subsequent analyses, and detailed information of them can be found in Supplementary Table 2. Intriguingly, the majority of them (66.7%, 6/9) were asymptomatic, except for one symptomatic case from China had an early AAO (26y) with initial symptom of hand tremor, and another symptomatic Japanese case had two long CGG expansions with the longer one comprising approximately 674 repeats, who also had an early AAO (27y) with initial symptom of muscle weakness.

Patients with negative pathological tests

Pathological tests were positive in almost all patients except for 6 cases (Supplementary Table 3). Briefly, 4 of them were from one article [78]. One patient (T5707 in [26]) suffering from consciousness disturbance and her disease duration was only 12 hours long. The last case harbored a heterozygous intermediate-length GGC repeat expansion and was previously diagnosed with PD [35]. Due to the dearth of acquired data, we failed to identify common characteristics in these patients with negative pathological results.

REFERENCES

[1] Tian Y, Wang JL, Huang W, Zeng S, Jiao B, Liu Z, *et al.* (2019). Expansion of Human-Specific GGC Repeat in Neuronal Intranuclear Inclusion Disease-Related Disorders. Am J Hum Genet, 105:166-176.

[2] Jiao B, Zhou L, Zhou Y, Weng L, Liao X, Tian Y, *et al.* (2020). Identification of expanded repeats in NOTCH2NLC in neurodegenerative dementias. Neurobiol Aging, 89:142 e141-142 e147.

[3] Yuan Y, Liu Z, Hou X, Li W, Ni J, Huang L, *et al.* (2020). Identification of GGC repeat expansion in the NOTCH2NLC gene in amyotrophic lateral sclerosis. Neurology, 95:e3394-e3405.

[4] Zhou X, Huang H, He R, Zeng S, Liu Z, Xu Q, *et al.* (2022). Clinical features and reclassification of essential tremor with NOTCH2NLC GGC repeat expansions based on a long-term follow-up. Eur J Neurol, 29:3600-3610.

[5] Deng J, Gu M, Miao Y, Yao S, Zhu M, Fang P, *et al.* (2019). Long-read sequencing identified repeat expansions in the 5'UTR of the NOTCH2NLC gene from Chinese patients with neuronal intranuclear inclusion disease. J Med Genet, 56:758-764.

[6] Deng J, Zhou B, Yu J, Han X, Fu J, Li X, *et al.* (2022). Genetic origin of sporadic cases and RNA toxicity in neuronal intranuclear inclusion disease. J Med Genet, 59:462-469.

[7] Yu J, Luan XH, Yu M, Zhang W, Lv H, Cao L, *et al.* (2021). GGC repeat expansions in NOTCH2NLC causing a phenotype of distal motor neuropathy and myopathy. Ann Clin Transl Neurol, 8:1330-1342.

[8] Zhao D, Zhu S, Xu Q, Deng J, Wang Z, Liu X (2021). Neuronal intranuclear inclusion disease presented with recurrent vestibular migraine-like attack: a case presentation. BMC Neurol, 21:334.

[9] Wang H, Yu J, Yu M, Deng J, Zhang W, Lv H, *et al.* (2021). GGC Repeat Expansion in the NOTCH2NLC Gene Is Associated With a Phenotype of Predominant Motor-Sensory and Autonomic Neuropathy. Front Genet, 12:694790.

[10] Yu J, Deng J, Guo X, Shan J, Luan X, Cao L, *et al.* (2021). The GGC repeat expansion in NOTCH2NLC is associated with oculopharyngodistal myopathy type 3. Brain, 144:1819-1832.

© 2024. Zeng T et al. Published online at http://www.aginganddisease.org/EN/10.14336/AD.2024.0131-1

[11] Wang ZY, Guo JJ, Wang M, Wang ZX, Hong DJ, Yu XF (2020). Adult-onset neuronal intranuclear inclusion disease mimicking Parkinson's disease in a Chinese patient: a case report and literature reviews. Neuro Endocrinol Lett, 41:155-161.
[12] Guo X, Wang Z, Li F (2022). Diffuse Cortical Injury and Basal Ganglia High Signals on Diffusion-Weighted Imaging in Neuronal Intranuclear Inclusion Disease. JAMA Neurol.
[13] Hong D, Wang H, Zhu M, Peng Y, Huang P, Zheng Y, *et al.* (2023). Subclinical peripheral neuropathy is common in neuronal intranuclear inclusion disease with dominant encephalopathy. Eur J Neurol, 30:527-537.
[14] Li F, Wang Q, Zhu Y, Xiao J, Gu M, Yu J, *et al.* (2022). Unraveling rare form of adult-onset NIID by characteristic brain MRI features: A single-center retrospective review. Front Neurol, 13:1085283.
[15] Wu W, Yu J, Qian X, Wang X, Xu Y, Wang Z, *et al.* (2022). Intermediate-length CGG repeat expansion in NOTCH2NLC is associated with pathologically confirmed Alzheimer's disease. Neurobiol Aging, 120:189-195.
[16] Liang H, Wang B, Li Q, Deng J, Wang L, Wang H, *et al.* (2020). Clinical and pathological features in adult-onset NIID patients with cortical enhancement. J Neurol, 267:3187-3198.
[17] Fang P, Yu Y, Yao S, Chen S, Zhu M, Chen Y, *et al.* (2020). Repeat expansion scanning of the NOTCH2NLC gene in patients with multiple system atrophy. Ann Clin Transl Neurol, 7:517-526.
[18] Wu C, Xiang H, Chen R, Zheng Y, Zhu M, Chen S, *et al.* (2022). Genetic spectrum in a cohort of patients with distal hereditary motor neuropathy. Ann Clin Transl Neurol, 9:633-643.
[19] Zhou Y, Huang P, Huang Z, Peng Y, Zheng Y, Yu Y, *et al.* (2022). Urine cytological study in patients with clinicopathologically confirmed neuronal intranuclear inclusion disease. Front Aging Neurosci, 14:977604.
[20] Zhang S, Gong Q, Wu D, Tian Y, Shen L, Lu J, *et al.* (2020). Genetic and Pathological Characteristic Patterns of a Family With Neuronal Intranuclear Inclusion Disease. J Neuropathol Exp Neurol, 79:1293-1302.
[21] Li M, Li K, Li X, Tian Y, Shen L, Wu G, *et al.* (2020). Multiple reversible encephalitic attacks: a rare manifestation of neuronal intranuclear inclusion disease. BMC Neurol, 20:125.
[22] Chen H, Lu L, Wang B, Cui G, Wang X, Wang Y, *et al.* (2020). Re-defining the clinicopathological spectrum of neuronal intranuclear inclusion disease. Ann Clin Transl Neurol, 7:1930-1941.
[23] Chen H, Lu L, Wang B, Hua X, Wan B, Sun M, *et al.* (2020). Essential tremor as the early symptom of NOTCH2NLC gene-related repeat expansion disorder. Brain, 143:e56.
[24] Huang Y, Jin G, Zhan QL, Tian Y, Shen L (2021). Adult-onset neuronal intranuclear inclusion disease, with both stroke-like onset and encephalitic attacks: a case report. BMC Neurol, 21:142.
[25] Deng WP, Yang Z, Huang XJ, Jiang JW, Luan XH, Cao L (2021). Case Report: Neuronal Intranuclear Inclusion Disease With Oromandibular Dystonia Onset. Front Neurol, 12:618595.
[26] Cao Y, Wu J, Yue Y, Zhang C, Liu S, Zhong P, *et al.* (2022). Expanding the clinical spectrum of adult-onset neuronal intranuclear inclusion disease. Acta Neurol Belg, 122:647-658.
[27] Cao Y, Tian W, Wu J, Song X, Cao L, Luan X (2022). DNA hypermethylation of NOTCH2NLC in neuronal intranuclear inclusion disease: a case-control study. J Neurol, 269:6049-6057.
[28] Cao Y, Tian W, Cao L, Lv W, Zheng L, Luan X (2022). Generation of an induced pluripotent stem cell JTUi005-A from a patient with neuronal intranuclear inclusion disease. Stem Cell Res, 65:102938.
[29] Yan Y, Cao L, Gu L, Zhang B, Xu C, Pu J, *et al.* (2021). Assessing the NOTCH2NLC GGC expansion in essential tremor patients from eastern China. Brain, 144:e1.
[30] Dong H, Ji G, Liu P, Li Y, Tian Y, Shen L, *et al.* (2020). A case of adult-onset neuronal intranuclear inclusion disease without abnormal high-intensity signal in the corticomedullary junction in diffusion-weighted imaging. Neurol Sci, 41:2653-2655.
[31] Ji G, Zhao Y, Zhang J, Dong H, Wu H, Chen X, *et al.* (2022). NOTCH2NLC-related oculopharyngodistal myopathy type 3 complicated with focal segmental glomerular sclerosis: a case report. BMC Neurol, 22:243.
[32] Guo JJ, Wang ZY, Wang M, Jiang ZZ, Yu XF (2020). Neuronal intranuclear inclusion disease mimicking acute cerebellitis: A case report. World J Clin Cases, 8:6122-6129.
[33] Zhang GJ, Wu D, Zhu YX, Ni HF, Zhang ZJ (2022). Clinicopathological features of neuronal intranuclear inclusion disease diagnosed by skin biopsy. Neurol Sci, 43:1809-1815.
[34] Shi CH, Fan Y, Yang J, Yuan YP, Shen S, Liu F, *et al.* (2021). NOTCH2NLC Intermediate-Length Repeat Expansions Are Associated with Parkinson Disease. Ann Neurol, 89:182-187.
[35] Fan Y, Liu F, Fan LY, Mao CY, Liu H, Zhang C, *et al.* (2021). Generation of an induced pluripotent stem cell line (ZZUi020-A) from a patient with Parkinson's disease harboring the intermediate-length GGC repeat expansions in the NOTCH2NLC gene. Stem Cell Res, 52:102257.
[36] Pang J, Yang J, Yuan Y, Gao Y, Shi C, Fan S, *et al.* (2021). The Value of NOTCH2NLC Gene Detection and Skin Biopsy in the Diagnosis of Neuronal Intranuclear Inclusion Disease. Front Neurol, 12:624321.
[37] Liu Y, Zeng L, Yuan Y, Wang Y, Chen K, Chen Y, *et al.* (2022). Case report: Two siblings with neuronal intranuclear inclusion disease exhibiting distinct clinicoradiological findings. Front Neurol, 13:1013213.
[38] Wang YC, Fan Y, Yu WK, Shen S, Li JD, Gao Y, *et al.* (2022). NOTCH2NLC expanded GGC repeats in patients with cerebral small vessel disease. Stroke Vasc Neurol.
[39] Li J, Fan Y, Fan L, Liu F, Hao X, Li M, *et al.* (2022). Generation of an induced pluripotent stem cell line (ZZUi036-A) derived from skin fibroblasts of a Neuronal intranuclear inclusion disease patient with GGC repeat expansion in the NOTCH2NLC gene. Stem Cell Res, 63:102844.
[40] Yang D, Cen Z, Wang L, Chen X, Liu P, Wang H, *et al.* (2022). Neuronal intranuclear inclusion disease tremor-dominant subtype: A mimicker of essential tremor. Eur J Neurol, 29:450-458.

[41] Liu P, Yang D, Zhang F, Chen S, Xie F, Luo Y, *et al.* (2022). The role of NOTCH2NLC in Parkinson's disease: A clinical, neuroimaging, and pathological study. Eur J Neurol, 29:1610-1618.
[42] Wang H, Feng F, Liu J, Deng J, Bai J, Zhang W, *et al.* (2022). Sporadic adult-onset neuronal intranuclear inclusion disease without high-intensity signal on DWI and T2WI: a case report. BMC Neurol, 22:150.
[43] Xie F, Hu X, Liu P, Zhang D (2022). A Case Report of Neuronal Intranuclear Inclusion Disease Presenting With Recurrent Migraine-Like Attacks and Cerebral Edema: A Mimicker of MELAS. Front Neurol, 13:837844.
[44] Liu C, Luan X, Liu X, Wang X, Cai X, Li T, *et al.* (2022). Characteristics of ocular findings of patients with neuronal intranuclear inclusion disease. Neurol Sci, 43:3231-3237.
[45] Zhang W, Ma J, Shi J, Huang S, Zhao R, Pang X, *et al.* (2022). GGC repeat expansions in NOTCH2NLC causing a phenotype of lower motor neuron syndrome. J Neurol, 269:4469-4477.
[46] Zhou Q, Tian M, Yang H, Luo YB (2022). Adult-Onset Neuronal Intranuclear Inclusion Disease with Mitochondrial Encephalomyopathy, Lactic Acidosis, and Stroke-Like (MELAS-like) Episode: A Case Report and Review of Literature. Brain Sci, 12.
[47] Su N, Mao HJ, Mao CH, Cui LY, Zhu YC, Zhou Y, *et al.* (2022). Recurrent headache and visual symptoms in a young man: a rare neuronal intranuclear inclusion disease case report. BMC Neurol, 22:401.
[48] Wu J, Li Q, Yi J (2022). Teaching NeuroImage: Occipital Cortical Enhancement During Encephalopathy-like Episode in Neuronal Intranuclear Inclusion Disease. Neurology, 99:964-965.
[49] Wu C, Wang M, Wang X, Li W, Li S, Chen B, *et al.* (2022). The genetic and phenotypic spectra of adult genetic leukoencephalopathies in a cohort of 309 patients. Brain.
[50] Gao X, Shao ZD, Zhu L (2022). Typical imaging manifestation of neuronal intranuclear inclusion disease in a man with unsteady gait: A case report. World J Clin Cases, 10:12388-12394.
[51] Zhao B, Yang M, Wang Z, Yang Q, Zhang Y, Qi X, *et al.* (2022). Clinical characteristics of two patients with neuronal intranuclear inclusion disease and literature review. Front Neurosci, 16:1056261.
[52] Lou Y, Yu JY, Shuai ZF, Zhao T, Wang YW, Liu XL (2022). Adult-onset neuronal nuclear inclusion disease presenting with mental and behavioral disorders: A case report and literature review. Aging Med (Milton), 5:297-302.
[53] Sone J, Mitsuhashi S, Fujita A, Mizuguchi T, Hamanaka K, Mori K, *et al.* (2019). Long-read sequencing identifies GGC repeat expansions in NOTCH2NLC associated with neuronal intranuclear inclusion disease. Nat Genet, 51:1215-1221.
[54] Mizutani K, Sakurai K, Uchida Y, Oguri T, Kato H, Yoshida M, *et al.* (2022). Absence of diffusion-weighted imaging abnormalities in a patient with neuronal intranuclear inclusion disease. Neurol Sci, 43:6551-6554.
[55] Ishiura H, Shibata S, Yoshimura J, Suzuki Y, Qu W, Doi K, *et al.* (2019). Noncoding CGG repeat expansions in neuronal intranuclear inclusion disease, oculopharyngodistal myopathy and an overlapping disease. Nat Genet, 51:1222-1232.
[56] Nakamura N, Tsunoda K, Mitsutake A, Shibata S, Mano T, Nagashima Y, *et al.* (2020). Clinical Characteristics of Neuronal Intranuclear Inclusion Disease-Related Retinopathy With CGG Repeat Expansions in the NOTCH2NLC Gene. Invest Ophthalmol Vis Sci, 61:27.
[57] Lim SY, Ishiura H, Ramli N, Shibata S, Almansour MA, Tan AH, *et al.* (2020). Adult-onset neuronal intranuclear inclusion disease mimicking Fragile X-associated tremor-ataxia syndrome in ethnic Chinese patients. Parkinsonism Relat Disord, 74:25-27.
[58] Okamura S, Takahashi M, Abe K, Inaba A, Sone J, Orimo S (2020). A case of neuronal intranuclear inclusion disease with recurrent vomiting and without apparent DWI abnormality for the first seven years. Heliyon, 6:e04675.
[59] Hayashi T, Katagiri S, Mizobuchi K, Yoshitake K, Kameya S, Matsuura T, *et al.* (2020). Heterozygous GGC repeat expansion of NOTCH2NLC in a patient with neuronal intranuclear inclusion disease and progressive retinal dystrophy. Ophthalmic Genet, 41:93-95.
[60] Tachi K, Takata T, Kume K, Sone J, Kobara H, Deguchi K, *et al.* (2021). Long-term MRI findings of adult-onset neuronal intranuclear inclusion disease. Clin Neurol Neurosurg, 201:106456.
[61] Okubo M, Doi H, Fukai R, Fujita A, Mitsuhashi S, Hashiguchi S, *et al.* (2019). GGC Repeat Expansion of NOTCH2NLC in Adult Patients with Leukoencephalopathy. Ann Neurol, 86:962-968.
[62] Ogasawara M, Iida A, Kumutpongpanich T, Ozaki A, Oya Y, Konishi H, *et al.* (2020). CGG expansion in NOTCH2NLC is associated with oculopharyngodistal myopathy with neurological manifestations. Acta Neuropathol Commun, 8:204.
[63] Ogasawara M, Eura N, Iida A, Kumutpongpanich T, Minami N, Nonaka I, *et al.* (2022). Intranuclear inclusions in muscle biopsy can differentiate oculopharyngodistal myopathy and oculopharyngeal muscular dystrophy. Acta Neuropathol Commun, 10:176.
[64] Ogasawara M, Eura N, Nagaoka U, Sato T, Arahata H, Hayashi T, *et al.* (2022). Intranuclear inclusions in skin biopsies are not limited to neuronal intranuclear inclusion disease but can also be seen in oculopharyngodistal myopathy. Neuropathol Appl Neurobiol, 48:e12787.
[65] Kikumoto M, Nezu T, Shiga Y, Motoda A, Toko M, Kurashige T, *et al.* (2021). Case of Neuronal Intranuclear Inclusion Disease With Dynamic Perfusion Changes Lacking Typical Signs on Diffusion-Weighted Imaging. Neurol Genet, 7:e601.
[66] Fukuda H, Yamaguchi D, Nyquist K, Yabuki Y, Miyatake S, Uchiyama Y, *et al.* (2021). Father-to-offspring transmission of extremely long NOTCH2NLC repeat expansions with contractions: genetic and epigenetic profiling with long-read sequencing. Clin Epigenetics, 13:204.
[67] Kameyama S, Mizuguchi T, Doi H, Koyano S, Okubo M, Tada M, *et al.* (2022). Patients with biallelic GGC repeat expansions in NOTCH2NLC exhibiting a typical neuronal intranuclear inclusion disease phenotype. Genomics, 114:110469.
[68] Miyamoto Y, Okazaki T, Watanabe K, Togawa M, Adachi T, Kato A, *et al.* (2023). First detailed case report of a pediatric patient with neuronal intranuclear inclusion disease diagnosed by NOTCH2NLC genetic testing. Brain Dev, 45:70-76.

© 2024. Zeng T et al. Published online at http://www.aginganddisease.org/EN/10.14336/AD.2024.0131-1

[69] Taneda T, Kanazawa M, Higuchi Y, Baba H, Isami A, Uemura M, *et al.* (2022). Neuronal Intranuclear Inclusion Disease Presenting with Voice Tremor. Mov Disord Clin Pract, 9:404-406.
[70] Tokimura R, Hashimoto Maeda M, Mitsutake A, Sakai S, Suzuki F, Sugasawa K, *et al.* (2022). Isolated Paravermal Hyperintensities in Neuronal Intranuclear Inclusion Disease. Neurology, 98:938-939.
[71] Yuan JH, Higuchi Y, Ando M, Matsuura E, Hashiguchi A, Yoshimura A, *et al.* (2022). Multi-type RFC1 repeat expansions as the most common cause of hereditary sensory and autonomic neuropathy. Front Neurol, 13:986504.
[72] Sugiyama A, Sone J, Kuwabara S (2022). Teaching NeuroImage: Paravermal Lesions in Neuronal Intranuclear Inclusion Disease. Neurology, 99:484-485.
[73] Orihara A, Miyakoshi N, Sunami Y, Kimura H, Nakata Y, Komori T, *et al.* (2022). A Case of Acute Reversible Encephalopathy with Neuronal Intranuclear Inclusion Disease Diagnosed by a Brain Biopsy: Inferring the Mechanism of Encephalopathy from Radiological and Histological Findings. Intern Med.
[74] Fukushima K, Hashimoto T, Yako T, Nakamura A, Oguchi K, Hayashi R, *et al.* (2022). Deep Brain Stimulation on Neuronal Intranuclear Inclusion Disease-Related Tremor: A Double-Edged Impact? Mov Disord Clin Pract, 9:983-986.
[75] Kurihara M, Komatsu H, Sengoku R, Shibukawa M, Morimoto S, Matsubara T, *et al.* (2023). CSF P-Tau181 and Other Biomarkers in Patients With Neuronal Intranuclear Inclusion Disease. Neurology, 100:e1009-e1019.
[76] Homma T, Nagaoka U, Nakata Y, Sone J, Funai A, Murayama A, *et al.* (2023). Neuropathological features of adult-onset neuronal intranuclear inclusion disease with fluid-attenuated inversion recovery high-intensity signals in the cerebellar paravermal area from an early stage: A case report. Clin Neuropathol, 42:66-73.
[77] Jung NY, Lee HJ, Mizuguchi T, Matsumoto N (2022). Genetic and Imaging Characteristics of a Family With Neuronal Intranuclear Inclusion Disease. J Clin Neurol, 18:358-360.
[78] Chen Z, Xu Z, Cheng Q, Tan YJ, Ong HL, Zhao Y, *et al.* (2020). Phenotypic bases of NOTCH2NLC GGC expansion positive neuronal intranuclear inclusion disease in a Southeast Asian cohort. Clin Genet, 98:274-281.
[79] Ng ASL, Lim WK, Xu Z, Ong HL, Tan YJ, Sim WY, *et al.* (2020). NOTCH2NLC GGC Repeat Expansions Are Associated with Sporadic Essential Tremor: Variable Disease Expressivity on Long-Term Follow-up. Ann Neurol, 88:614-618.
[80] Ma D, Tan YJ, Ng ASL, Ong HL, Sim W, Lim WK, *et al.* (2020). Association of NOTCH2NLC Repeat Expansions With Parkinson Disease. JAMA Neurol, 77:1559-1563.
[81] Liao YC, Chang FP, Huang HW, Chen TB, Chou YT, Hsu SL, *et al.* (2022). GGC Repeat Expansion of NOTCH2NLC in Taiwanese Patients With Inherited Neuropathies. Neurology, 98:e199-e206.
[82] Hsia Y, Cheng CY, Tang SC, Lin CW (2021). Peculiar pattern of retinopathy in adult-onset neuronal intranuclear inclusion disease. J Formos Med Assoc, 120:1793-1795.
[83] Liu YH, Chou YT, Chang FP, Lee WJ, Guo YC, Chou CT, *et al.* (2022). Neuronal intranuclear inclusion disease in patients with adult-onset non-vascular leukoencephalopathy. Brain, 145:3010-3021.
[84] Chen Z, Yan Yau W, Jaunmuktane Z, Tucci A, Sivakumar P, Gagliano Taliun SA, *et al.* (2020). Neuronal intranuclear inclusion disease is genetically heterogeneous. Ann Clin Transl Neurol, 7:1716-1725.
[85] Yau WY, Vandrovcova J, Sullivan R, Chen Z, Zecchinelli A, Cilia R, *et al.* (2021). Low Prevalence of NOTCH2NLC GGC Repeat Expansion in White Patients with Movement Disorders. Mov Disord, 36:251-255.

© 2024. Zeng T et al. Published online at http://www.aginganddisease.org/EN/10.14336/AD.2024.0131-1
